# Thermodynamic Study of Alkylsilane and Alkylsiloxane-Based Ionic Liquids

**DOI:** 10.1021/acs.jpcb.3c08333

**Published:** 2024-04-04

**Authors:** Rodrigo M.A. Silva, Hadrián Montes-Campos, Ana I.M.C. Lobo Ferreira, Eduards Bakis, Luís M.N.B.F. Santos

**Affiliations:** † CIQUP, Institute of Molecular Sciences (IMS), Department of Chemistry and Biochemistry, Faculty of Science, University of Porto, Rua do Campo Alegre, Porto 4169-007, Portugal; ‡ Faculty of Chemistry, University of Latvia, Jelgavas 1, Riga LV-1004, Latvia

## Abstract

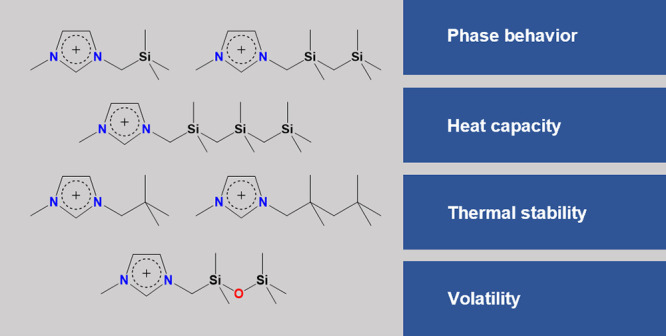

The thermodynamic properties of ionic liquids (ILs) bearing alkylsilane and alkylsiloxane chains, as well as their carbon-based analogs, were investigated. Effects such as the replacement of carbon atoms by silicon atoms, the introduction of a siloxane linkage, and the length of the alkylsilane chain were explored. Differential scanning calorimetry (DSC) and thermogravimetric analysis (TGA) were used to study the thermal and phase behavior (glass transition temperature, melting point, enthalpy and entropy of fusion, and thermal stability). Heat capacity was obtained by high-precision drop calorimetry and differential scanning microcalorimetry. The volatility and cohesive energy of these ILs were investigated via the Knudsen effusion method coupled with a quartz crystal microbalance (KEQCM). Gas phase energetics and structure were also studied to obtain the gas phase heat capacity as well as the energy profile associated with the rotation of the IL side chain. The computational study suggested the existence of an intramolecular interaction in the alkylsiloxane-based IL. The obtained glass transition temperatures seem to follow the trend of chain flexibility. An increase of the alkylsilane chain leads to a seemingly linear increase in molar heat capacity. A regular increment of 30 J·K^–1^·mol^–1^ in the molar heat capacity was found for the replacement of carbon by silicon in the IL alkyl chain. The alkylsilane series was revealed to be slightly more volatile than its carbon-based analogs. A further increase in volatility was found for the alkylsiloxane-based IL, which is likely related to the decrease of the cohesive energy due to the existence of an intramolecular interaction between the siloxane linkage and the imidazolium headgroup. The use of Si in the IL structure is a suitable way to significantly reduce the IL’s viscosity while preserving its large liquid range (low melting point and high thermal stability) and low volatilities.

## 1 Introduction

Recently, the thermodynamic study of ionic liquid (IL) series has attracted great attention due to academic motivation, as well as their potential application and unique functionalities.^1−3^ This arises as a consequence of their properties, such as low flammability and volatility, high thermal and electrochemical stability, low melting point, and large liquid range. However, given the dominant Coulombic interactions, ILs tend to have moderate to high viscosities, a property that has been regarded as a major drawback to their application. Researchers have therefore investigated ways of modifying ILs with the aim of reducing their viscosity while maintaining their other properties. One of the options that have been regarded as an alternative is the use of ILs that bear alkylsilane or alkylsiloxane chains (SiILs) as a replacement of the typical alkyl-based chain.

This strategy was first approached by Shirota and Castner^4^ when the authors investigated the effect of inserting a silicon atom in the cation structure. For this, they compared the dynamic properties of 1-alkyl-3-methylimidazolium ILs in which the alkyl chain was a trimethylsilylmethyl (SiC) or a neopentyl (Np) group. The only difference between these groups is that the quaternary carbon in the Np group is replaced by a silicon atom in the SiC group. The authors found that for the [NTf_2_]-based ILs, the viscosity of the IL with the [(SiC)C_1_im] cation was 1.6 times lower than the viscosity of the IL with the [(Np)C_1_im] cation. This effect was more pronounced for the [BF_4_]-based ILs, with viscosity being reduced by a factor of 7.4. The reduction in viscosity found in these ILs has been correlated with two major factors: (i) the higher flexibility of the Si-containing chains due to the lowering of the energetic barrier associated with the rotation of the Si–C bond and (ii) the higher polarizability of Si-containing groups, which contributes to weaker cation–anion interactions.^4−9^

Later, the same authors investigated the properties of an IL containing a (1,1,3,3,3-pentamethyldisiloxaneyl)methyl chain (SiOSiC).^5^ They found that although the [(SiOSiC)C_1_im][NTf_2_] IL contains a bulkier cation than [(SiC)C_1_im][NTf_2_], the viscosities of these ILs do not differ significantly. The reduced viscosity of the IL containing the [(SiOSiC)C_1_im] cation is associated, again, with the higher chain flexibility due to the presence of a siloxane linkage. Niedermeyer et al.^10^ performed an extended quantum chemical and experimental study on [(SiOSiC)C_1_im][Cl]. They verified that, because of the siloxane linkage, the IL chain is also able to shield certain positions from engaging in H-bonding, therefore reducing the overall strength of the H-bonding network.

Recently, Bakis et al.^11^ synthesized and studied several Si-containing ILs, as well as some of their carbon-based analogs. The authors found that although the introduction of Si in the cation increases its weight, it also reduces its density, in accordance with the expansion in the cation size provoked by the longer Si–C bond. The authors also found that, analogously to what happens in *n*-alkyl-substituted ILs, the density of the IL is reduced as the alkylsilane chain becomes longer. Furthermore, two [(SiOSiC)C_1_im] analogs were synthesized, namely, those bearing 2,2,4,4-tetramethyl-2,4-disilapentyl (SiCSiC) and 2,2,4,4-tetramethylpentyl (Me_4_C_5_) chains. Regarding their dynamics, it was revealed that the viscosity of [(Me_4_C_5_)C_1_im][NTf_2_] is 6.9 and 13.1 times higher than that of [(SiCSiC)C_1_im][NTf_2_] and [(SiOSiC)C_1_im][NTf_2_], respectively

Bakis et al.^11^ also studied the solubility of argon in these ILs. They verified that larger cations, those that bear alkylsilane or alkylsiloxane chains, have a higher argon solubility. For the cations with shorter chains (Np or SiC), the use of Si in the structure does not produce a measurable difference in the solubility of argon. This property was revealed to be more dramatically affected by the choice of larger and more flexible anions.

Si-containing ILs have also been investigated for diverse applications such as gas adsorption and separation,^12^ dye-sensitized solar cells,^13,14^ surfactants,^15^ and polymerization.^16^ Although these ILs have revealed interesting and promising properties, studies on their phase behavior are very scarce, and to the best of our knowledge, no studies have reported properties such as heat capacity or thermal stability, which are of high relevance not only from the academic but also from the industrial standpoint.

**Figure 1:**
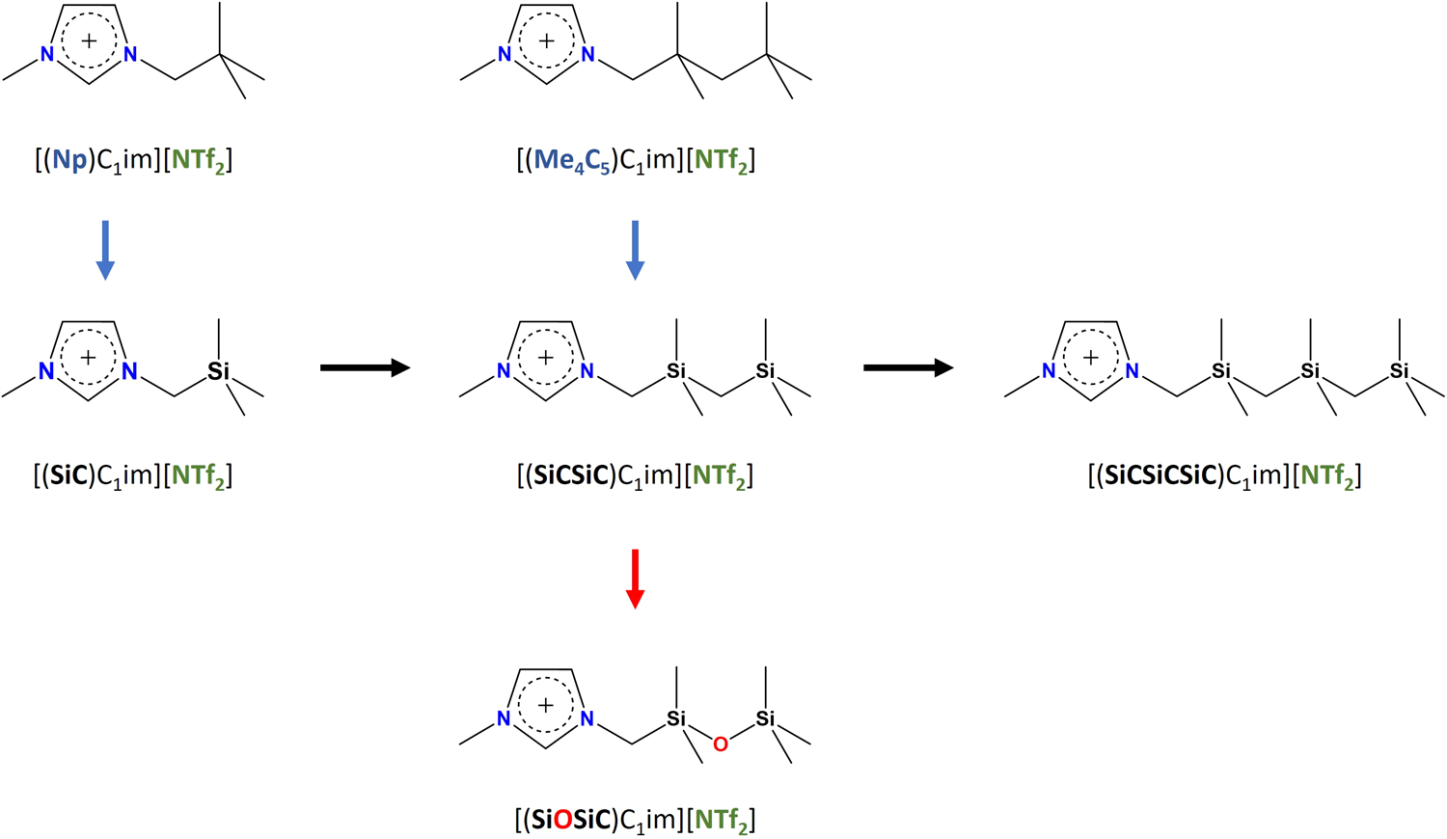
Structure and nomenclature of the cations that compose the studied ILs and schematic representation of the strategy followed to investigate the structural changes in the IL series.

In this work, we investigated the phase behavior, volatility, heat capacity, and thermal stability of several SiILs, as well as some of their carbon-based analogs, with the aim of understanding the effect that introducing Si in the IL structure has on these properties. This is the first time that these properties have been investigated on this type of ILs. The study of these new type of ILs can improve their potential applicability due to their enhanced properties and functionalities. A scheme containing the structures of the cations of the investigated ILs and the adopted nomenclature for each IL is presented in Figure 1.

The scheme in Figure 1 also depicts the different effects that we aimed to evaluate with this work: the effect of replacing the quaternary carbon atoms with silicon atoms (blue arrows), the effect of the length of the alkylsilane chain (black arrows), and the effect of the siloxane linkage (red arrow).

## 2 Methods

### 2.1 Materials

The ILs studied in this work were synthesized in accordance with the procedure described in a previous work.^11^ Before experiments, the ILs were degassed and dried in a vacuum (*p* < 10 Pa) at *T* = 333 K, with continuous stirring, during a minimum of 48 h to reduce the content of volatile impurities. The relative atomic masses used were those recommended by the IUPAC Commission in 2016.^17^ The studied ILs are presented in Table 1. It was found by Karl Fischer analysis that the water content was below 100 ppm in all the samples. NMR data of the ILs are provided in a previous publication.^11^

**Table 1:** Nomenclature and Molar Mass for the Studied Ionic Liquids

ionic liquid	chemical formula	CAS number	*M*/g·mol^–1^
1-methyl-3-(trimethylsilylmethyl)imidazolium bis(trifluoromethanesulfonyl)imide	[(SiC)C_1_im][NTf_2_]	871127-68-3	449.4702
1-methyl-3-(2,2,4,4-tetramethyl-2,4-disilapentyl)imidazolium bis(trifluoromethanesulfonyl)imide	[(SiCSiC)C_1_im][NTf_2_]		521.6508
1-(2,2,4,4,6,6-hexamethyl-2,4,6-trisilaheptyl)-3-methylimidazolium bis(trifluoromethanesulfonyl)imide	[(SiCSiCSiC)C_1_im][NTf_2_]		593.8314
1-methyl-3-((1,1,3,3,3-pentamethyldisiloxaneyl)methyl)imidazolium bis(trifluoromethanesulfonyl)imide	[(SiOSiC)C_1_im][NTf_2_]	936638-34-5	523.6236
1-methyl-3-neopentylimidazolium bis(trifluoromethanesulfonyl)imide	[(Np)C_1_im][NTf_2_]	871127-69-4	433.3958
1-methyl-3-(2,2,4,4-tetramethylpentyl)imidazolium bis(trifluoromethanesulfonyl)imide	[(Me_4_C_5_)C_1_im][NTf_2_]		489.5020

### 2.2 Solid–Liquid Equilibrium

The phase behavior of the investigated ILs was determined via differential scanning calorimetry (DSC) using a commercial DSC (PerkinElmer, model Pyris Diamond). A constant flow (20 mL·min^–1^) of highly pure gaseous nitrogen (99.999%) was used as protective gas. IL samples (3 to 14 mg) were hermetically sealed in 50 *μ*L aluminum crucibles under a dry nitrogen atmosphere. In this work, the melting point was taken as the onset temperature of the peak associated with the melting process. The glass transition temperature was taken as the temperature at the midpoint of the heat capacity change of the glass transition.

Temperature and heat flux scales of the DSC were calibrated by determining the melting point and enthalpy of fusion of several reference and recommended materials such as benzoic acid,^18^*o*-terphenyl,^19^ 1,3,5-triphenylbenzene,^19^ perylene,^18,19^ 1,3-difluorobenzene,^18^ and indium.^18^

The IL sample was in the liquid state at the beginning of all DSC experiments. With the intent of promoting glass formation, the first step consisted of a fast cooling at 40 K·min^–1^ down to 183 K. Afterward, the temperature was increased at 5 K·min^–1^ until glass transition and cold crystallization were observed. In the case of the occurrence of cold crystallization, a fast temperature decrease (until 183 K) would follow, and then a heating scan at 5 K·min^–1^ would be performed. This cycling procedure was repeated until no glass transition, cold crystallization, or phase reorganization was detected, ensuring that the whole sample was in the crystalline state. The melting points and enthalpies of fusion were determined using a scanning rate of 5 K·min^–1^.

### 2.3 Thermal Stability

The thermal stability of the ILs was studied by means of thermogravimetric analysis (TGA) using a NETZSCH thermomicrobalance (model TG 209 F1 Iris). The experiments were performed under a nitrogen atmosphere (99.999%). Flows of 10 and 40 mL·min^–1^ were used as protective and purge gas, respectively. During experiments, the samples were held in 25 μL aluminum crucibles and were heated between 303 and 743 K. The evaluation of the thermal stability was performed using four distinct scanning rates (*β* = 0.8, 2, 5, and 10 K·min^–1^), and the decomposition temperature at null scanning rate, *T*_d_ (*β* = 0 K·min^–1^), was taken based on the linear extrapolation of the onset of the TGA curve, *T*_d_, as a function of *β*^1/3^ (extrapolation methodology based in the model that provided the best linear description of the scanning rate dependence). Additional information, data analysis, and raw data are available as SI.

### 2.4 High-Precision Heat Capacity Measurements

#### 2.4.1 High-Precision Drop Calorimetry

A high-precision drop-type heat conduction differential calorimeter was used to determine the heat capacity of the ILs at *T* = 298.15 K. The apparatus has been described in previous publications^20−23^ and was calibrated using sapphire (NBS, SRM 720, *α*-Al_2_O_3_). The accuracy of the measurements performed with this calorimeter is better than 0.5%. Raw experimental data are presented in Section 4 of the SI.

#### 2.4.2 Heat Conduction Differential Scanning Microcalorimetry

The heat capacity as a function of temperature (between *T* = 283 and 333 K) was measured using a customized version of a heat conduction differential scanning microcalorimeter (micro DSC III from SETARAM). The customization of this calorimeter is described ahead. During the experiments, the sample was kept in a 1 cm^3^ Hastelloy C276 cell hermetically sealed with a Viton O-ring. The apparatus operated using the incremental step method. Regarding experimental details, temperature steps (Δ*T*_step_) of 10.0 K were done at a heating rate (*β*) of 0.30 K·min^–1^ followed by an isothermal delay (*t*_isothermal_) of 3600 s. With this methodology, the overall uncertainty of the heat capacity measurements was estimated to be lower than 0.75%. The obtained heat capacities at the different experimental temperatures are presented in Section 5 of the SI.

##### 2.4.2.1 Customization of the SETARAM Micro DSC III

The differential scanning microcalorimeter used in this work is a customized version of a SETARAM micro DSC III. The homemade customization consisted of the modification of the temperature control system by the use of a new heat pump/exchanger system (model: LL-210-24-00, Laird Thermal Systems), reinforcement of the thermal insulation of the calorimeter, and replacement of the original preamplification board by a customized low noise preamplifier (gain of 1000×), originally built in the Thermochemistry Laboratory of Lund University. Furthermore, a new temperature control module was built and incorporated into the calorimeter. The temperature control module is depicted in Figure S1 of the SI. The temperature sensors of this calorimeter (Pt200) were calibrated by means of comparison against a Standard Reference Platinum Thermometer (FLUKE 5626, probe #2329). Performance tests revealed that, in all cases, the results obtained with the customized version of the HC-DSC are in excellent agreement with results obtained by other authors with high-precision techniques, namely, adiabatic and drop calorimetry. Further details on the refurbishment and testing of the HC-DSC are provided in Section 1 of the SI.

### 2.5 Vapor Pressure Measurements

The vapor pressure of each IL was measured as a function of temperature by means of a Knudsen effusion apparatus coupled with a quartz crystal microbalance (KEQCM). Both the apparatus and the adopted methodology have been described in previous literature.^24−27^ This apparatus combines two mass-loss detection techniques (gravimetric and quartz crystal microbalance). Through this methodology, it is possible to measure the vapor pressure of the sample at several different temperatures during the same experiment, making the duration of the experiments shorter. The rate of change of the crystal’s frequency of resonance, corrected to a background effect, , is related to the vapor pressure, *p*(*T*), through the following equation:
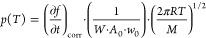
1where *W* is the sensitivity coefficient of the quartz crystal microbalance, *A*_0_ is the area of the orifice of the Knudsen cell, *w*_0_ is the transmission probability factor, *R* is the gas constant (*R* = 8.314462618 J·K^–1^·mol^–1^), *T* is the experimental temperature, and *M* is the molar mass of the studied compound.

### 2.6 Quantum Chemical Calculations

We performed quantum density functional theory (DFT) to find the optimized geometry of the IL pair in the gas phase as well as their thermodynamic properties, such as the heat capacity. All calculations were carried out using Gaussian 16^28^ with the B3LYP exchange and correlation functional and the 6-311G(d,p) basis set. The integration grid was set to ultrafine. To find the configuration of minimum energy, 24 different starting configurations were generated for each IL, with the anion placed around different regions of the cation, similarly to previous reports.^29,30^ After the minimization, a frequency analysis was performed for all the configurations to obtain the thermodynamic properties at the temperature of *T* = 298.15 K. Finally, the configuration with lower free energy was chosen as the most representative of the thermodynamic ensemble. A scaling factor of 0.985 was used.^31^ The resulting configurations for all the minimizations are displayed in the SI (Section 7) as well as the energy difference with respect to the configuration of minimum energy.

## 3 Results and Discussion

### 3.1 Quantum Chemical Analysis of the Configurations

**Figure 2:**
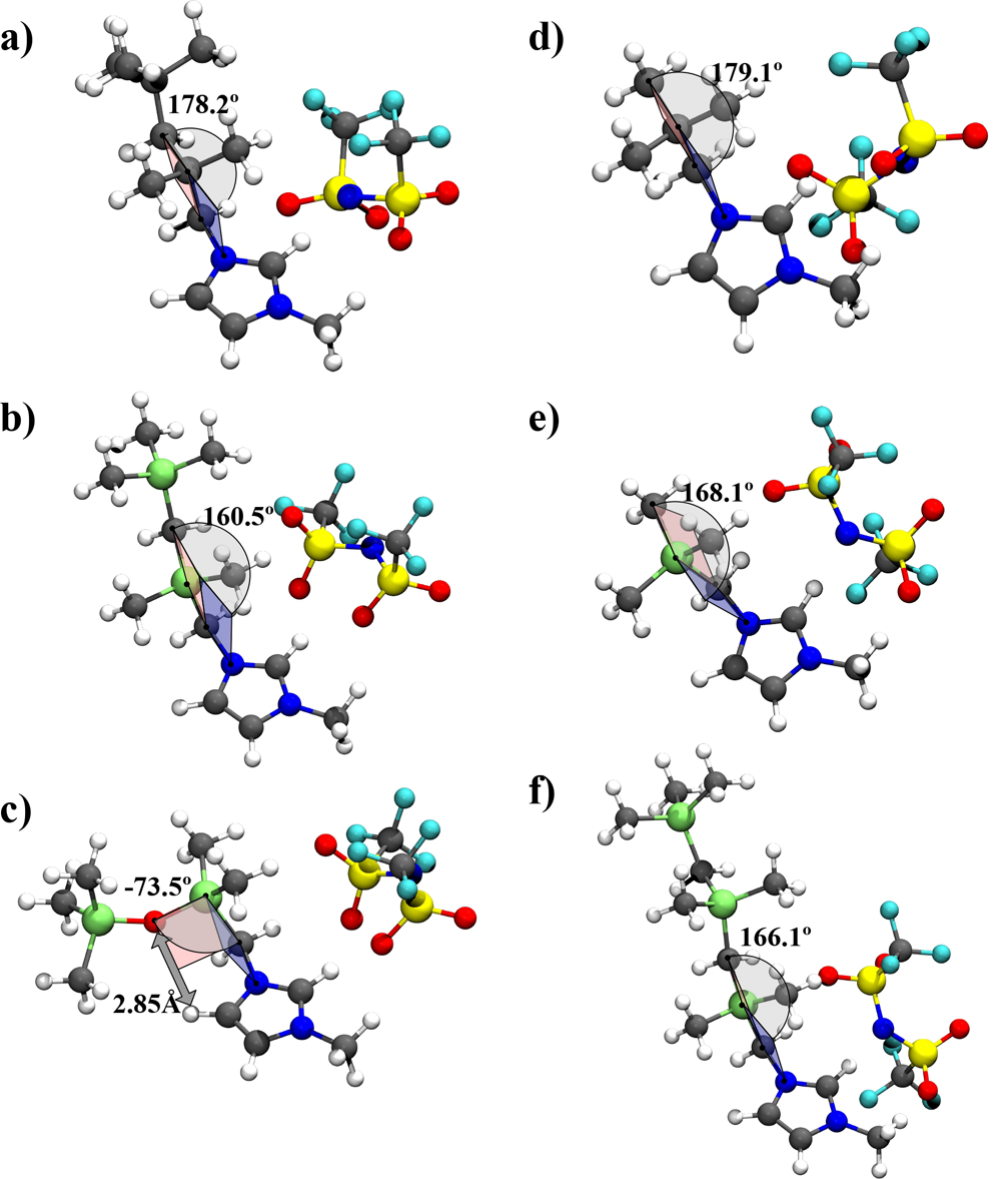
Representation of the configurations of minimum energy from the quantum calculations. (a) [(Me_4_C_5_)C_1_im][NTf_2_]; (b) [(SiCSiC)C_1_im][NTf_2_]; (c) [(SiOSiC)C_1_im][NTf_2_]; (d) [(SiC)C_1_im][NTf_2_]; (e) [(SiC)C_1_im][NTf_2_]; and (f) [(SiCSiCSiC)C_1_im][NTf_2_].

The analysis of the configurations of minimum energy (which are reproduced in Figure 2) shows that, for all the configurations, the anion is placed coordinating with the most acidic hydrogen of the imidazolium ring (the one bonded to the C2 carbon). Moreover, all but the alkylsiloxane cation have their chain in its most extended configuration. On the other hand, the alkylsiloxane cation has its tail coiled in the direction of the C5 carbon. This seems to indicate that a hydrogen bond is formed between the oxygen atom of the alkylsiloxane chain and the hydrogen atom bonded to the C5 carbon. A similar observation has been done by Niedermeyer et al.^10^ for the crystalline phase of [(SiOSiC)C_1_im][Cl]. Furthermore, it is widely accepted that a similar interaction occurs in ether-based ILs, where the oxygen atoms in the chain interact with the acidic hydrogen atoms in the imidazolium group.^32−36^ Some differences were observed in the conformation of the anion, with some of them coordinating with the nitrogen atom and others with the oxygen atoms. However, the energy difference between these conformers seems to be very small (around 0.5 kJ·mol^–1^ as can be seen from comparing, for example, configurations 9 and 10 of [(Me_4_C_5_)C_1_im][NTf_2_]).

**Figure 3:**
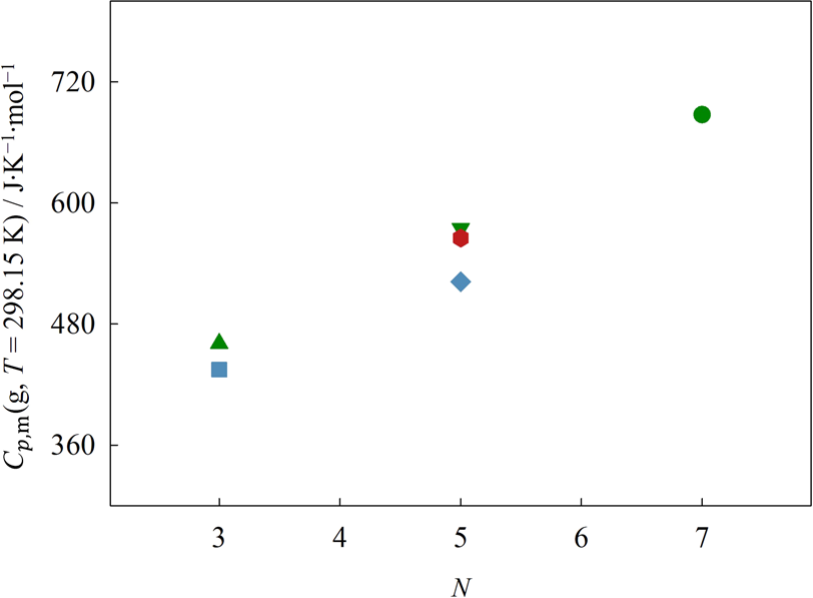
Plot of the molar heat capacity, *C*_*p*,m_, of the gas phase, at *T* = 298.15 K, as a function of the total number of atoms on the cation side chain backbone (*N*). Green symbols: ▲ [(SiC)C_1_im][NTf_2_], ▼ [(SiCSiC)C_1_im][NTf_2_], ●[(SiCSiCSiC)C_1_im][NTf_2_]; blue symbols: ■ [(Np)C_1_im][NTf_2_], ◆ [(Me_4_C_5_)C_1_im][NTf_2_]; red symbols: ⬢ [(SiOSiC)C_1_im][NTf_2_]. Plotted values correspond to quantum calculations with the B3LYP method (6-311G(d,p) basis set; scaling factor of 0.985).

The isobaric molar heat capacities of the configurations of minimum energy, calculated using the B3LYP method, are presented in Table 2. Furthermore, they are represented as a function of the total number of atoms on the cation side chain backbone in Figure 3.

**Table 2:** Isobaric Molar Heat Capacity, *C*_*p*,m_, at *T* = 298.15 K of the Configurations of Minimum Energy Calculated by the B3LYP Method (6-311G(d,p) Basis Set; Scaling Factor of 0.985) in the Gas phase

ionic liquid	*C*_*p*,m_ (g, *T* = 298.15 K)/J·K^–1^·mol^–1^
[(SiC)C_1_im][NTf_2_]	460.7
[(SiCSiC)C_1_im][NTf_2_]	574.8
[(SiCSiCSiC)C_1_im][NTf_2_]	687.5
[(SiOSiC)C_1_im][NTf_2_]	565.2
[(Np)C_1_im][NTf_2_]	434.9
[(Me_4_C_5_)C_1_im][NTf_2_]	521.8

An approximately linear increase of heat capacity with the chain length is observed for the alkylsilane family. Moreover, a decrease in heat capacity is observed when the Si atoms are replaced with C atoms. This is related to the Si–C bond being weaker than the C–C and therefore having a lower frequency. The decrease in frequency of the normal modes when replacing C by Si atoms consequently decreases the vibrational temperature, which in turn leads to a higher contribution of the corresponding vibrational frequency to the heat capacity. This occurs because, in this temperature range, the ratio between the temperature and vibrational temperature is significantly less than the unity.^37^ A small decrease in heat capacity is also observed when replacing the alkylsilane chain with an alkylsiloxane one, which is due to the decrease in the number of atoms and may also be associated with the formation of the hydrogen bond that reduces the degrees of freedom of the alkylsiloxane chain.

**Figure 4:**
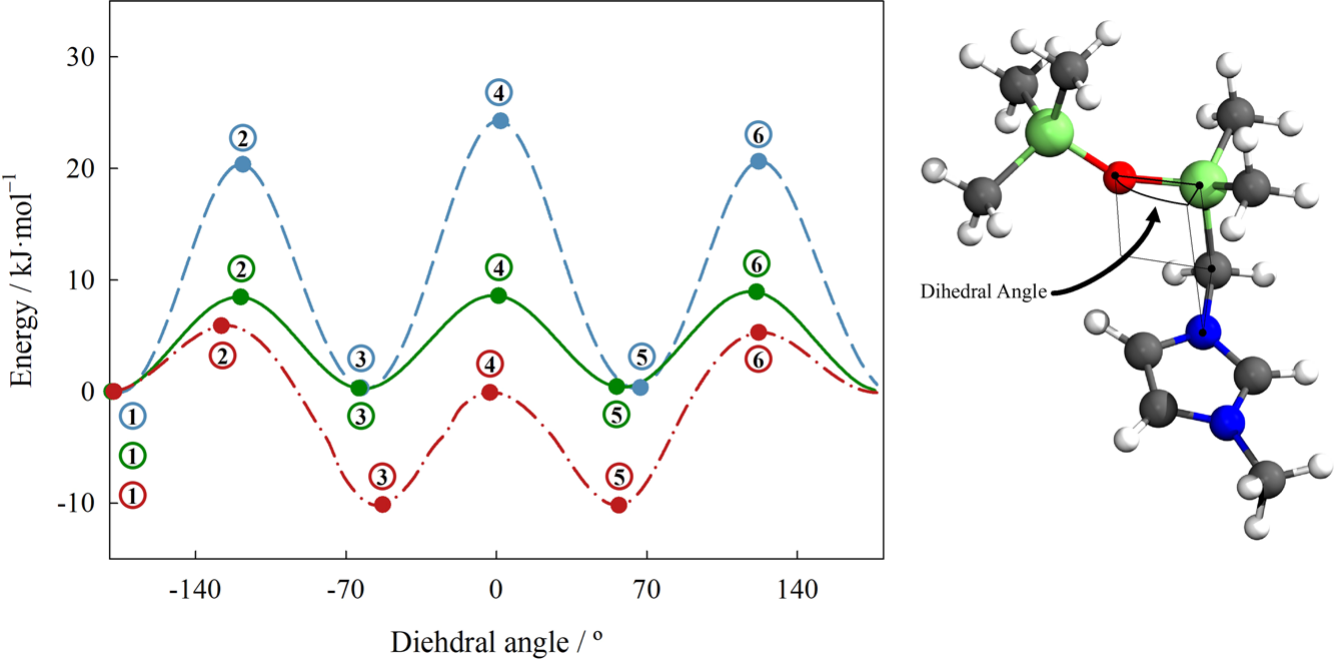
Energy as a function of the dihedral angle. Energy is measured with respect to the configuration with the fully extended tail (180°) using B3LYP/6-311G(d,p). [(SiCSiC)C_1_im][NTf_2_] (green solid line), [(Me_4_C_5_)C_1_im][NTf_2_] (blue dashed line), [(SiOSiC)C_1_im][NTf_2_] (red dash–dot–dash line).

To characterize the flexibility of the different chains, as well as the possible hydrogen bonding between the side chain and the aromatic ring, we performed a scan of the energetics of the rotation of the chain for the three cations with five atoms in their cation side chain backbone (*N* = 5). To reduce the number of degrees of freedom of the system, as well as steric effects, the anion was omitted during these calculations. The scan was performed by modifying the dihedral angle, shown in Figure 2, in steps of 5° while relaxing all the other internal coordinates of the system. The results are presented in Figure 4.

The energies of the configurations marked in Figure 4 are presented in Table 3. It can be seen that both the alkylsilane and the alkyl chains show a profile with a periodicity of around 120° that is characteristic of tetrahedral geometries. The three minima of the profile have approximately the same energy, which is symptomatic that there are no relevant interactions between the tail and the imidazolium ring as has been previously reported for other imidazolium-based ionic liquids.^38^ Moreover, the energy barrier between the minima is greatly reduced for the alkylsilane chain. An analogous observation was done by Phillipi et al.^7^ while studying the energetics of the [(Np)C_1_im] and [(SiC)C_1_im] cations.

**Table 3:** Energy of the Configurations Marked in Figure 4^a^

		energy/kJ·mol^–1^	
configuration	[(SiCSiC)C_1_im][NTf_2_]	[(Me_4_C_5_)C_1_im][NTf_2_]	[(SiOSiC)C_1_im][NTf_2_]
1	0	0	0
2	8.48	20.35	5.90
3	0.28	0.32	–10.14
4	8.59	24.26	–0.08
5	0.42	0.34	–10.19
6	8.94	20.62	5.29

a Calculated using B3LYP/6-311G(d,p).

On the other hand, whereas the alkylsiloxane presents the same three minima located at approximately the same dihedral angle, the configurations at ±60° have much lower energy than the fully extended one. This change in energy is in the typical range for hydrogen bonds^39^ and strongly suggests the existence of hydrogen bonding between the oxygen of the alkylsiloxane tail and the acidic hydrogen of the imidazolium ring. The change in energy is greater than 10 kJ·mol^–1^, much higher than the thermal energy at room temperature (2.5 kJ·mol^–1^) and, therefore, this interaction should also be relevant in the condensed phase. The presence of this hydrogen bond should also reduce the cohesive energy of the liquid phase due to the blocking of an interaction site and the reduced surface area of the molecule with respect to the stretched configuration.^40^

### 3.2 Thermal Behavior

The phase behavior of the ILs with alkylsilane and alkylsiloxane chains, as well as their carbon-based chain analogs, was investigated through DSC. The thermograms obtained for the studied ILs are available as SI (Section 2). The determined glass transition temperature and melting point (*T*_g_ and *T*_m_, respectively) are presented in Table 4 together with the glass transition temperature/melting point ratios (*T*_g_/*T*_m_). The standard (*p*^o^ = 10^5^ Pa) molar isobaric heat capacity change at the glass transition (Δ_gl_^1^*C*_*p*,m_^o^) and the standard molar enthalpy and entropy of fusion (Δ_cr_^1^*H*_m_^o^ and Δ_cr_^1^*S*_m_^o^, respectively), are presented in Table 5.

**Table 4:** Glass Transition Temperature (*T*_g_), Melting Point (*T*_m_), and Glass Transition Temperature/Melting Point Ratio (*T*_g_/*T*_m_) for the Studied Ionic Liquids^a^

ionic liquid	*T*_g_/K	*T*_m_/K	*T*_g_/*T*_m_
[(SiC)C_1_im][NTf_2_]	204		
	201^5^		
	205^16^		
	201^41^		
[(SiCSiC)C_1_im][NTf_2_]	206	285.7	0.71
[(SiCSiCSiC)C_1_im][NTf_2_]	203	293.3	0.69
[(SiOSiC)C_1_im][NTf_2_]	198	252.8	0.78
	197^5^		
[(Np)C_1_im][NTf_2_]	210		
	203^41^		
[(Me_4_C_5_)C_1_im][NTf_2_]	215		

a The standard uncertainties were estimated to be ±1 K for *T*_g_ and ±0.5 K for *T*_m_ and include the calibration uncertainty.

**Table 5:** Standard Molar Heat Capacity Change at the Glass Transition (Δ_gl_^1^*C*_*p*,m_^o^), Standard Molar Melting Enthalpy (Δ_cr_^1^*H*_m_^o^), and Standard Molar Melting Entropy (Δ_cr_^l^*S*_m_^o^) for the Studied Ionic Liquids^a^

ionic liquid	Δ_gl_^1^*C*_*p*,m_^o^/J·K^–1^· mol^–1^	Δ_cr_^l^*H*_m_^o^ (*T*_m_)/kJ·mol^–1^	Δ_cr_^1^*S*_m_^o^ (*T*_m_)/J·K^–1^·mol^–1^
[(SiC)C_1_im][NTf_2_]	112		
[(SiCSiC)C_1_im][NTf_2_]	148	24.3	84.9
[(SiCSiCSiC)C_1_im][NTf_2_]	153	33.4	113.8
[(SiOSiC)C_1_im][NTf_2_]	120	14.3	56.6
[(Np)C_1_im][NTf_2_]	125		
[(Me_4_C_5_)C_1_im][NTf_2_]	123		

a Standard pressure (*p*^o^ = 10^5^ Pa). The standard uncertainties were estimated to be ±1 K for *T*_g_ and ±0.5 K for *T*_m_. The standard uncertainties for Δ_cr_^l^*H*_m_^o^(*T*_m_) were estimated to be ±1.0 kJ·mol^–1^ and ±4.0 J·K^–1^·mol^–1^ for Δ_cr_^1^*S*_m_^o^(*T*_m_). For Δ_gl_^1^*C*_*p*,m_^o^, the standard uncertainty was estimated to be ±10 J·K^–1^· mol^–1^. All these uncertainties include the calibration uncertainty.

**Figure 5:**
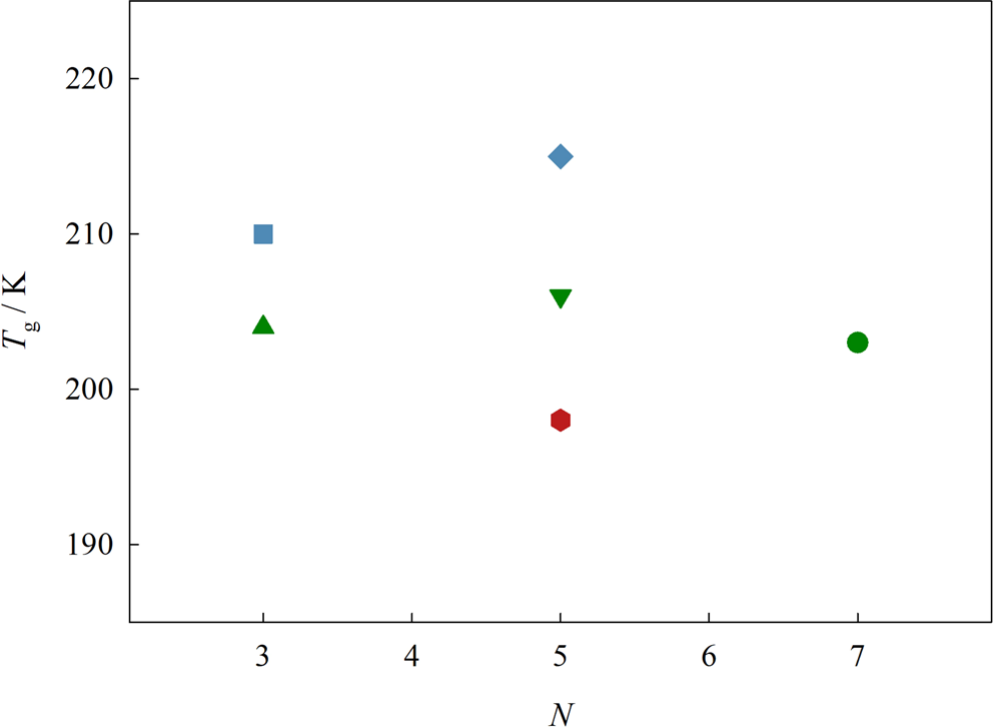
Glass transition temperature, *T*_g_, as a function of the number of atoms on the cation side chain backbone. Green symbols: ▲ [(SiC)C_1_im][NTf_2_], ▼ [(SiCSiC)C_1_im][NTf_2_], ●[(SiCSiCSiC)C_1_im][NTf_2_]; blue symbols: ■ [(Np)C_1_im][NTf_2_], ◆ [(Me_4_C_5_)C_1_im][NTf_2_]; red symbols: ⬢ [(SiOSiC)C_1_im][NTf_2_].

In this work, the glassy state was successfully achieved for all the studied ILs. The glass transition temperatures determined in this work for [(SiC)C_1_im][NTf_2_] and [(SiOSiC)C_1_im][NTf_2_] are in excellent agreement with those determined by Shirota et al.,^5^ Kaestner et al.,^16^ and Chung et al.^41^ However, the glass transition temperature we determined for [(Np)C_1_im][NTf_2_] is significantly higher than the one reported by Chung et al.^41^ The values obtained for the glass transition temperature of the studied ILs are presented as a function of the number of atoms in the backbone of the cation side chain in Figure 5.

It was found that the glass transition temperature of the ILs that have an alkylsilane chain is lower than that of their carbon-based chain analogs. This effect seems to be enhanced by the introduction of an oxygen atom in the backbone (forming a siloxane linkage, Si–O–Si) because the *T*_g_ of the IL with an alkylsiloxane chain is lower than that of both its alkylsilane and carbon-based chain analogs. When comparing the glass transition temperature of carbon-based chain ILs with those of ether-based chain ILs, Philippi et al.^35^ also found a lower *T*_g_ for the ILs in which the chain contains oxygen atoms. The trend of *T*_g_ seems to follow the trend of chain flexibility decrease: Me_4_C_5_ > SiCSiC > SiOSiC.

In this work, the occurrence of melting was only successfully detected for three out of the six studied ILs: [(SiCSiC)C_1_im][NTf_2_], [(SiCSiCSiC)C_1_im][NTf_2_], and [(SiOSiC)C_1_im][NTf_2_] ILs. Although melting data were only obtained for two of the alkylsilane-based ILs, the results reveal that an increment in the size of the alkylsilane chain produces an increase in the melting point and in the enthalpy and entropy of fusion. This increase should be related to an intensification of the dispersive chain–chain interactions due to the lengthening of the IL chain, similarly to what has been reported for long *n*-alkyl-based ILs.^42−44^ The *T*_g_/*T*_m_ ratios calculated for the alkylsilane-based ILs are in good agreement with the usual value found for ILs^45^ and with that predicted by the Beaman–Kauzmann rule for one-component glass-forming liquids (*T*_g_/*T*_m_ ≈ 2/3).^46−48^ However, a much higher value of the *T*_g_/*T*_m_ ratio was found for the alkylsiloxane-based IL. Nonetheless, similar *T*_g_/*T*_m_ ratio values have also been reported for both alkyl^44,49^ and ether-based ILs.^33,35^

Previously, when investigating the phase behavior of the [(SiOSiC)C_1_im][NTf_2_] IL, Shirota et al.^5^ only detected the occurrence of glass transition. In fact, during our experiments, no crystallization peak was observed for this IL. Upon heating of the quenched sample, glass transition would be observed followed by a very small endothermic peak around *T* = 253 K. To further explore this phenomenon, an isotherm with a duration of 1 h was performed at *T* = 243 K followed by rapid cooling of the sample. Upon reheating, no glass transition was found, and an endothermic peak occurred at *T* = 252.8 K, which was associated with a melting process. These observations reveal that the [(SiOSiC)C_1_im][NTf_2_] IL has slow crystallization dynamics. Because of its slow-paced occurrence, the heat of crystallization is released in small quantities over time, leading to the absence of a clear crystallization peak.

When comparing the melting points of the ILs with alkylsilane and alkylsiloxane chains, it is noticeable that the IL that has an alkylsiloxane chain has a lower melting point. A lowering of the melting point of ILs upon insertion of oxygen in the cation chain was also observed by Phillipi et al.^35^ In the past, Niedermeyer et al.^10^ synthesized [(SiOSiC)C_1_im][Cl] and determined its crystal structure through X-ray crystallography. In the crystal structure, it is possible to notice that the oxygen atom in the Si–O–Si linkage seems to interact with the H atom in the C2 position of the imidazolium ring, which should reduce the interactions of the acidic H with the anion. A similar interaction could be present in crystalline [(SiOSiC)C_1_im][NTf_2_], explaining the observed reduction in Δ_cr_^l^*H*_m_^o^.

The thermal stability of the ILs was studied by thermogravimetric analysis (TGA). The obtained thermograms (represented as mass percentage as a function of temperature) are presented in the SI (Section 3). The temperatures of decomposition, taken as the onset of the TGA curve, obtained at the different scanning rates are also presented in the SI (Table S5). The temperatures of decomposition extrapolated to null scanning rate are presented in Table 6.

**Table 6:** Extrapolated Temperature of Decomposition of the Studied ILs at Null Scanning Rate, *T*_d_ (*β* = 0 K·min^–1^)^a^

ionic liquid	*T*_d_ (*β* = 0 K·min^–1^)/K
[(SiC)C_1_im][NTf_2_]	579
[(SiCSiC)C_1_im][NTf_2_]	589
[(SiCSiCSiC)C_1_im][NTf_2_]	592
[(SiOSiC)C_1_im][NTf_2_]	486
[(Np)C_1_im][NTf_2_]	579
[(Me_4_C_5_)C_1_im][NTf_2_]	585

a The uncertainty for *T*_d_ (*β* = 0 K·min^–1^) was estimated to be ±5 K.

For all studied samples except [(SiOSiC)C_1_im][NTf_2_], a one-step decomposition was found. In the case of [(SiOSiC)C_1_im][NTf_2_], a complex decomposition was found, occurring in more than one step. Although we have not looked into the mechanism, this distinct behavior should arise because of the presence of the siloxane linkage in the IL, especially when one considers that its alkylsilane analog (which only differs by the oxygen atom) decomposes in one step. The onset temperatures of decomposition, at the different scanning rates, were found to be systematically higher for the SiILs when compared to their carbon-based analogs. However, the linear extrapolation of the onset temperature as a function of *β*^1/3^ (Table 5) reveals that there is no significant difference between the *T*_d_ of SiILs and carbon-based ILs when compounds with the same number of atoms on the backbone of the side chain of the cation are considered.

It also seems that, as the alkyl or alkylsilane chains become longer, the thermal stability of the IL slightly increases. Interestingly, the trend of *T*_d_ for the [C_*n*_C_*m*_im][NTf_2_] IL series as a function of the length of the alkyl side chain of the cation was reported to present a nonmonotonous behavior.^50,51^

### 3.3 Heat Capacity

**Figure 6:**
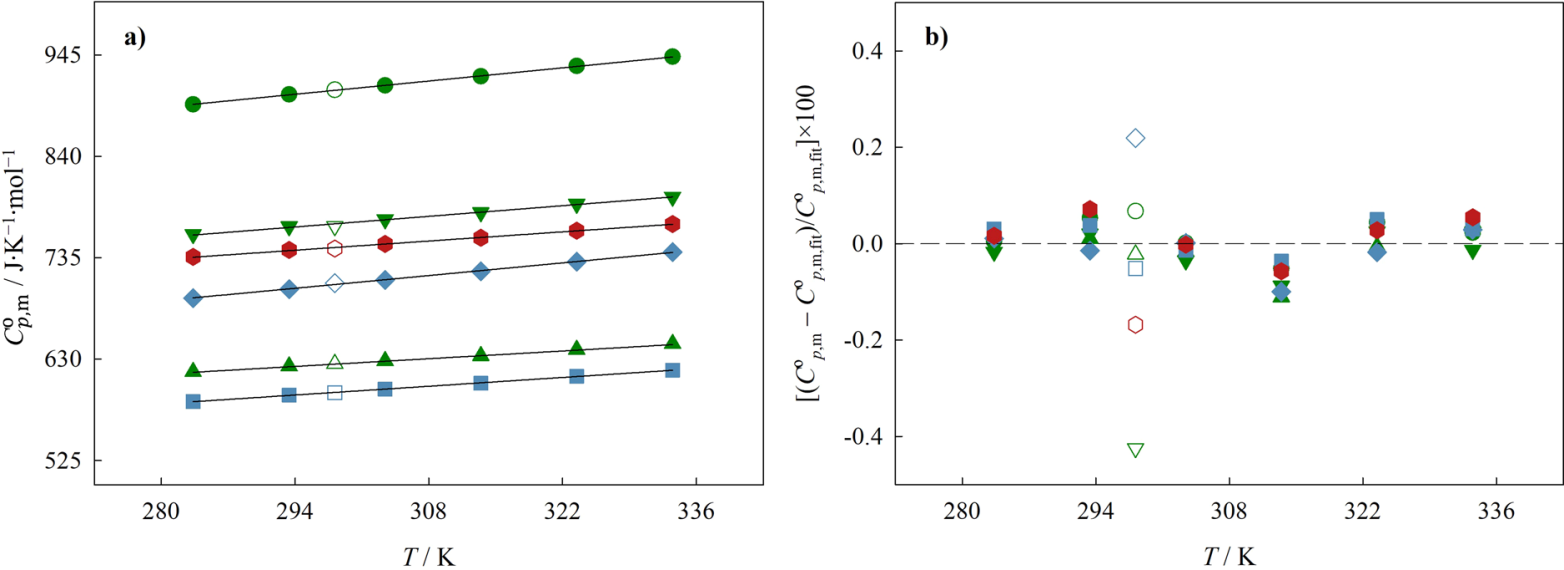
(a) Plot of the standard molar isobaric heat capacity, *C*_*p*,m_^o^, of the liquid phase as a function of temperature for the studied ionic liquids. (b) Plot of the relative residuals of the linear fit of the standard molar isobaric heat capacity. Green symbols: ▲ [(SiC)C_1_im][NTf_2_], ▼ [(SiCSiC)C_1_im][NTf_2_], ●[(SiCSiCSiC)C_1_im][NTf_2_]; blue symbols: ■ [(Np)C_1_im][NTf_2_], ◆ [(Me_4_C_5_)C_1_im][NTf_2_]; red symbols: ⬢ [(SiOSiC)C_1_im][NTf_2_]. Filled symbols are HC-DSC data, and empty symbols are drop calorimetry data. Error bars are smaller than the symbols.

The isobaric heat capacity of the liquid phase of the ILs was determined through high-precision drop calorimetry for the temperature of 298.15 K and through high-precision differential scanning microcalorimetry (HC-DSC) between 283 and 333 K. The heat capacities obtained with both techniques are represented as a function of temperature in Figure 6. The heat capacities obtained with HC-DSC are provided at the different experimental temperatures in Table S8.

For the results obtained by HC-DSC, the dependence of the heat capacity with temperature was fitted to the following equation:
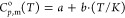
2

The *a* and *b* parameters obtained for all ILs are presented in Table 7. The values of standard specific, volumetric, and molar isobaric heat capacity (*C*_*p*,m_^o^/(*V*_m_), and *C*_*p*,m_^o^, respectively) at *T* = 298.15 K for the liquid phase of the studied ILs, determined by both high-precision drop calorimetry and HC-DSC, are presented in Table 8.

**Table 7:** Parameters Obtained from the Linear Fitting of the Molar Heat Capacity, as a Function of Temperature, for the Liquid Phase of the Studied Ionic Liquids^a^

ionic liquid	*T*_range_/K	*a*/J·K^–1^·mol^–1^	*b*/J·K^–2^·mol^–1^	*s*_r_ (%)
[(SiC)C_1_im][NTf_2_]	283–333	453.9 ± 2.8	0.575 ± 0.009	0.06
[(SiCSiC)C_1_im][NTf_2_]	283–333	540.8 ± 2.7	0.772 ± 0.009	0.05
[(SiCSiCSiC)C_1_im][NTf_2_]	283–333	615.8 ± 2.8	0.981 ± 0.009	0.04
[(Np)C_1_im][NTf_2_]	283–333	403.0 ± 1.7	0.646 ± 0.005	0.04
[(Me_4_C_5_)C_1_im][NTf_2_]	283–333	424.9 ± 2.7	0.947 ± 0.009	0.05
[(SiOSiC)C_1_im][NTf_2_]	283–333	545.2 ± 2.8	0.673 ± 0.009	0.06

a , in which *n* is the number of fitted data points and *m* is the number of independent adjustable parameters (here *m* = 2).

**Table 8:** Standard Volumetric (*C*_*p*,m_^o^/*V*_m_) and Molar (*C*_*p*,m_^o^) Isobaric Heat Capacities at *T* = 298.15 K for the Liquid Phase of the Studied Ionic Liquids (HC-DSC and Drop Calorimetry)^a^

	*C*_*p*,m_^o^/*V*_m_/J·K^–1^·cm^–3^^b^	*C*_*p*,m_^o^/J·K^–1^·mol^–1^	*C*_*p*,m_^o^/*V*_m_/J·K^–1^·cm^–3^^b^	*C*_*p*,m_^o^/J·K^–1^·mol^–1^
ionic liquid	HC-DSC		drop calorimetry	
[(SiC)C_1_im][NTf_2_]	1.011 ± 0.008	625.3 ± 4.7	1.011 ± 0.003	625.2 ± 1.9
[(SiCSiC)C_1_im][NTf_2_]	1.137 ± 0.009	771.0 ± 5.8	1.132 ± 0.003	767.7 ± 2.3
[(SiCSiCSiC)C_1_im][NTf_2_]	1.259 ± 0.009	908.3 ± 6.8	1.260 ± 0.004	908.9 ± 2.7
[(Np)C_1_im][NTf_2_]	0.974 ± 0.007	595.6 ± 4.5	0.973 ± 0.003	595.3 ± 1.8
[(Me_4_C_5_)C_1_im][NTf_2_]	1.101 ± 0.008	707.2 ± 5.3	1.104 ± 0.003	708.8 ± 2.1
[(SiOSiC)C_1_im][NTf_2_]	1.101 ± 0.008	745.9 ± 5.6	1.099 ± 0.003	744.6 ± 2.3

a Standard pressure (*p*^o^ = 10^5^ Pa). The combined expanded uncertainty (0.95 level of confidence, *k* = 2) of the heat capacity is *U*_c_ (*C*_*p*,m_^o^) = 0.075·*C*_*p*,m_^o^ for HC-DSC and *U*_c_ (*C*_*p*,m_^o^) = 0.030·*C*_*p*,m_^o^ for drop calorimetry.

b Calculated using the molar volumes reported by Bakis et al.^11^

By analyzing the *b* coefficients in Table 7, it is noticeable that the heat capacity dependence on temperature is larger for the ILs with carbon-based chains. Furthermore, when comparing the ILs with the same number of backbone atoms (*N* = 5), i.e., Me_4_C_5_, SiCSiC, and SiOSiC, the heat capacity dependence on temperature decreases in the following order: Me_4_C_5_ > SiCSiC > SiOSiC. This trend can be rationalized through the saturation of the hindered rotation energy levels. As has been mentioned, the insertion of Si atoms on the IL chain lowers the energy barriers associated with the rotation.^7^ It is then reasonable to assume that, in the studied temperature interval, the ILs with lower rotational energy barriers are closer to the classical limit when compared with the ILs bearing carbon-based chain, and so their contribution to the heat capacity increase is smaller.

**Figure 7:**
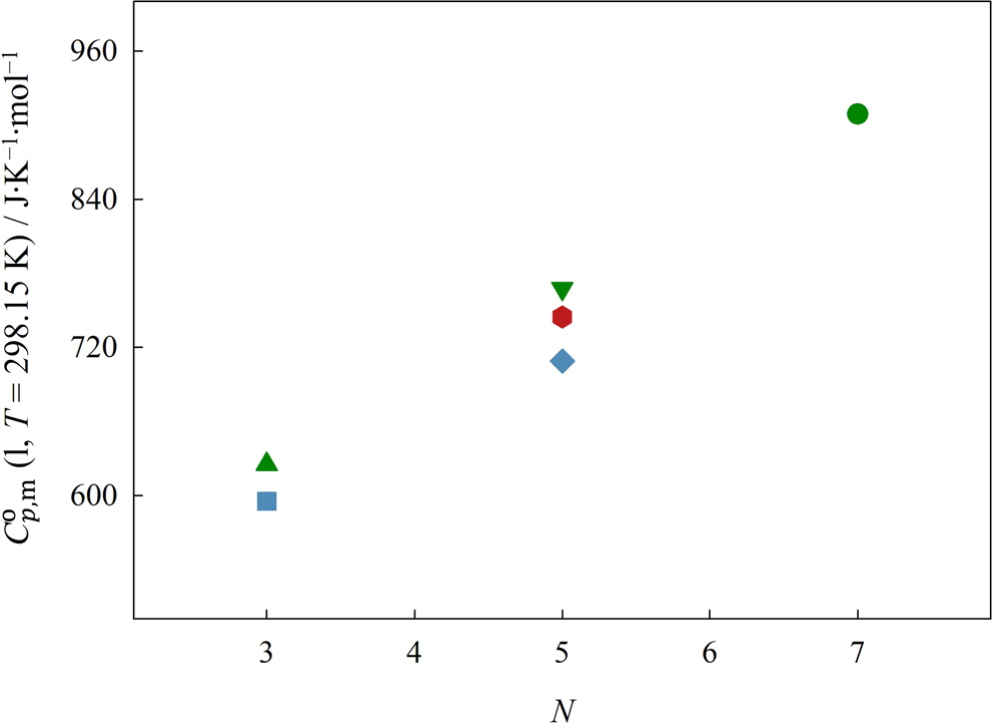
Plot of the standard molar heat capacity, *C*_*p*,m_^o^, of the liquid phase at *T* = 298.15 K as a function of the total number of atoms on the backbone of the cation side chain. Green symbols: ▲ [(SiC)C_1_im][NTf_2_], ▼ [(SiCSiC)C_1_im][NTf_2_], ●[(SiCSiCSiC)C_1_im][NTf_2_]; blue symbols: ■ [(Np)C_1_im][NTf_2_], ◆ [(Me_4_C_5_)C_1_im][NTf_2_]; red symbols: ⬢ [(SiOSiC)C_1_im][NTf_2_]. Plotted values correspond to drop calorimetry data. Error bars are smaller than the symbols.

For all ILs, the values obtained for the standard molar heat capacity at *T* = 298.15 K by HC-DSC and drop calorimetry are in mutual agreement. The values of standard isobaric molar heat capacity, *C*_*p*,m_^o^ (*T* = 298.15 K), obtained with drop calorimetry for the liquid phase of the ILs are presented in Figure 7 as a function of the total number of atoms on the backbone of the cation side chain.

The trends found in Figure 7 are identical to those found for the standard molar isobaric heat capacity of the gas phase in Figure 3. The results revealed that the molar heat capacities of the ILs that contain alkylsilane groups are larger than those of their carbon-based analogs. Moreover, the difference between the molar heat capacities of [(Np)C_1_im][NTf_2_] and [(SiC)C_1_im][NTf_2_] is around 30 J·K^–1^·mol^–1^, and the difference between the molar heat capacities of [(Me_4_C_5_)C_1_im][NTf_2_] and [(SiCSiC)C_1_im][NTf_2_] is approximately 60 J·K^–1^·mol^–1^. In the first instance, a quaternary carbon atom is replaced by a silicon atom in the IL’s chain, while in the second case, two quaternary carbon atoms are replaced. This suggests that, for each carbon atom which is replaced by a silicon atom in the IL’s chain, there is an increase in the molar heat capacity of 30 J·K^–1^·mol^–1^. This observation is likely due to the weaker nature of the Si–C bond when compared to the C–C bond, as discussed regarding the standard molar isobaric heat capacity of the gas phase. When comparing the molar heat capacities of the [(SiCSiC)C_1_im][NTf_2_] and [(SiOSiC)C_1_im][NTf_2_] ILs, one notices that the latter is smaller by about 13 J·K^–1^·mol^–1^. This decrease in molar heat capacity should be associated with the smaller number of energy-storing modes on the [(SiOSiC)C_1_im][NTf_2_] IL given that when the carbon is replaced with oxygen in the IL chain, there is a net loss of two hydrogen atoms in the structure. It should be noted that the liquid heat capacity of [(Np)C_1_im][NTf_2_] (*C*_*p*,m_^o^ (*T* = 298.15 K) = 595.3 ± 1.8 J·K^–1^·mol^–1^) is identical to those of [C_5_C_1_im][NTf_2_] (*C*_*p*,m_^o^ (*T* = 298.15 K) = 595.6 ± 0.5 J·K^–1^·mol^–1^)^52^ and [C_3_C_3_im][NTf_2_] (*C*_*p*,m_^o^ (*T* = 298.15 K) = 594.52 ± 0.49 J·K^–1^·mol^–1^)^53^ and that the heat capacity of [(Me_4_C_5_)C_1_im][NTf_2_] (*C*_*p*,m_^o^ (*T* = 298.15 K) = 708.8 ± 2.1 J·K^–1^·mol^–1^) is similar to that of [C_5_C_5_im][NTf_2_] (*C*_*p*,m_^o^ (*T* = 298.15 K) = 718.19 ± 0.71 J·K^–1^·mol^–1^)^53^.

By plotting the molar heat capacity of the ILs as a function of the number of alkyl, −CH_2_C(CH_3_)_2_–, or alkylsilane, −CH_2_Si(CH_3_)_2_–, segments, intercepts of 482 and 484 J·K^–1^·mol^–1^ are found, respectively. This value is relatively close to the molar heat capacity of the [C_1_C_1_im][NTf_2_] IL (*C*_*p*,m_^o^ (*T* = 298.15 K) = 472.33 ± 0.46 J·K^–1^·mol^–1^^53^).

This set of results reveals that the molar heat capacity of ILs containing alkylsilane chains in the liquid phase is nearly additive and that the prediction of the heat capacity (of the liquid phase, at *T* = 298.15 K) of other alkylsilane-based ILs might be possible using the simple group contribution models like that proposed by Gardas and Coutinho.^54^ One example could be the rule of
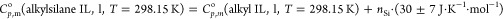
3where *n*_Si_ is the number of C atoms in the IL that have been replaced by Si atoms.

### 3.4 Vapor Pressure Measurements

**Figure 8:**
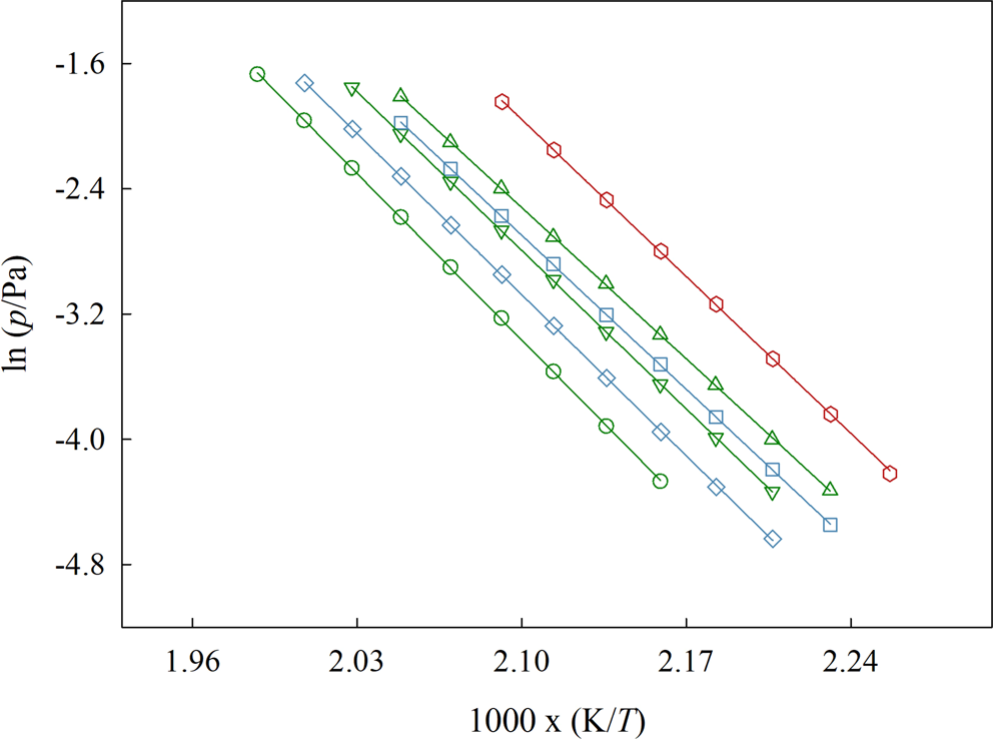
Graphical representation of ln(*p*/Pa) = *f*[(1/*T*)/K^–1^] for each of the studied ionic liquids. Green symbols: △ [(SiC)C_1_im][NTf_2_], ▽ [(SiCSiC)C_1_im][NTf_2_], ○ [(SiCSiCSiC)C_1_im][NTf_2_]; blue symbols: □ [(Np)C_1_im][NTf_2_], ◇ [(Me_4_C_5_)C_1_im][NTf_2_]; red symbols: ⬡ [(SiOSiC)C_1_im][NTf_2_].

The volatility of the studied ILs was evaluated by means of a Knudsen effusion apparatus coupled with a quartz crystal microbalance. The vapor pressures of the studied ILs at the different experimental temperatures are presented in Table S9. Figure 8 contains the representation of ln(*p*/Pa) = *f*[(1/*T*)/K^–1^] for each of the studied ILs. The experimental results were fitted to the Clarke and Glew equation, truncated on the second term of Δ_1_^g^*C*_*p*,m_^o^.^55^
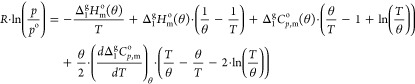
4where *p* is the experimental pressure, *p*^o^ is the reference pressure (*p*^o^ = 10^5^ Pa), *θ* is a selected reference temperature, and *T* is the experimental temperature. Δ_1_^g^*G*_m_^o^(*θ*) is the standard Gibbs energy of vaporization at the selected reference temperature, and Δ_1_^g^*H*_m_^o^(*θ*) is the standard enthalpy of vaporization at the selected reference temperature. Δ_1_^g^*C*_*p*,m_^o^(*θ*) is the difference between the heat capacities of the liquid and gas phase at the selected reference temperature. The term Δ_1_^g^*C*_*p*,m_^o^ and its temperature derivative were imposed as fixed in the fitting procedure. Δ_1_^g^*C*_*p*,m_^o^(*θ*) was calculated at the different reference temperatures through extrapolation of the Δ_1_^g^*C*_*p*,m_^o^(*T*) function, obtained through eq 2, using the parameters of Table 7, along with the computed gas phase heat capacity (details provided in Section 6 of the SI). In this work, two reference temperatures were used: *θ* = 460 and 298.15 K. The use of 460 K as reference temperature is preferred because, being closer to the mean experimental temperature, it reduces the contribution of Δ_1_^g^*C*_*p*, m_^o^ in the determination of the standard molar properties of vaporization. The parameters obtained for the fitting of the Clarke and Glew equation to the experimental data are presented in Table S10.

The values obtained for Δ_1_^g^*G*_m_^o^, Δ_1_^g^*H*_m_^o^, and Δ_1_^g^*S*_m_^o^ at the experimental and at the two reference temperatures, by the fitting of eq 4, are presented in Table 9 and are related to each other through the following equation:
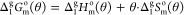
5

**Table 9:** Standard Molar Gibbs Energies (Δ_1_^g^*G*_m_^o^), Enthalpies (Δ_1_^g^*H*_m_^o^), and Entropies (Δ_1_^g^*S*_m_^o^) of Vaporization for the Studied Ionic Liquids at the Mean Experimental Temperature and at the Reference Temperatures (*θ* = 298.15 and 460 K)^a^

θ/K	Δ_1_^g^*G*_m_^o^/kJ·mol^–1^	Δ_1_^g^*H*_m_^o^/kJ·mol^–1^	Δ_1_^g^*S*_m_^o^/J·K^–1^·mol^–1^
[(SiC)C_1_im][NTf_2_]			
468.19^b^	56.5 ± 0.2	114.6 ± 0.3	124.1 ± 0.6
460	57.6 ± 0.2	115.3 ± 0.3	125.7 ± 0.6
298.15	81.8 ± 0.5	136.4 ± 1.7	183.1 ± 4.6
[(SiCSiC)C_1_im][NTf_2_]			
473.18^b^	57.0 ± 0.2	120.2 ± 0.2	133.5 ± 0.4
460	58.8 ± 0.2	121.6 ± 0.2	136.6 ± 0.5
298.15	85.6 ± 0.5	146.7 ± 1.8	205.2 ± 4.6
[(SiCSiCSiC)C_1_im][NTf_2_]			
483.16^b^	57.9 ± 0.2	126.1 ± 0.3	141.1 ± 0.6
460	61.2 ± 0.2	129.0 ± 0.4	147.2 ± 0.7
298.15	90.3 ± 0.5	157.2 ± 1.9	224.6 ± 4.9
[(Np)C_1_im][NTf_2_]			
468.17^b^	57.3 ± 0.2	116.8 ± 0.2	127.1 ± 0.4
460	58.3 ± 0.2	117.6 ± 0.2	128.9 ± 0.4
298.15	83.2 ± 0.5	138.9 ± 1.7	186.9 ± 4.5
[(Me_4_C_5_)C_1_im][NTf_2_]			
478.12^b^	57.5 ± 0.2	122.5 ± 0.3	135.9 ± 0.6
460	60.0 ± 0.2	124.9 ± 0.4	140.9 ± 0.7
298.15	87.7 ± 0.5	150.6 ± 1.8	210.7 ± 4.8
[(SiOSiC)C_1_im][NTf_2_]			
460.63^b^	55.5 ± 0.2	119.5 ± 0.5	139.0 ± 1.1
460	55.6 ± 0.2	119.5 ± 0.5	139.1 ± 1.1
298.15	82.0 ± 0.5	141.5 ± 1.7	199.5 ± 4.5

a Standard pressure (*p*^o^ = 10^5^ Pa). The standard uncertainties were calculated considering the uncertainties of the Clarke and Glew fitting coefficients and by applying the propagation of uncertainty to eq 5. The standard uncertainties associated with Δ_1_^g^*G*_m_^o^(*θ*) at ⟨*T*⟩ and at *θ* = 460 K were derived from the error of pressure. The standard uncertainties associated with Δ_1_^g^*G*_m_^o^(*θ*) at *θ* = 298.15 K were derived from the combination of the estimated errors for Δ_1_^g^*H*_m_^o^(*θ*) and Δ_1_^g^*S*_m_^o^(*θ*). An uncertainty of ±10 J·K^–1^·mol^–1^ was considered for Δ_1_^g^*C*_*p*,m_^o^. The standard uncertainty of the temperature is *u*(*T*) = 0.02 K.

b Mean experimental temperature.

**Figure 9:**
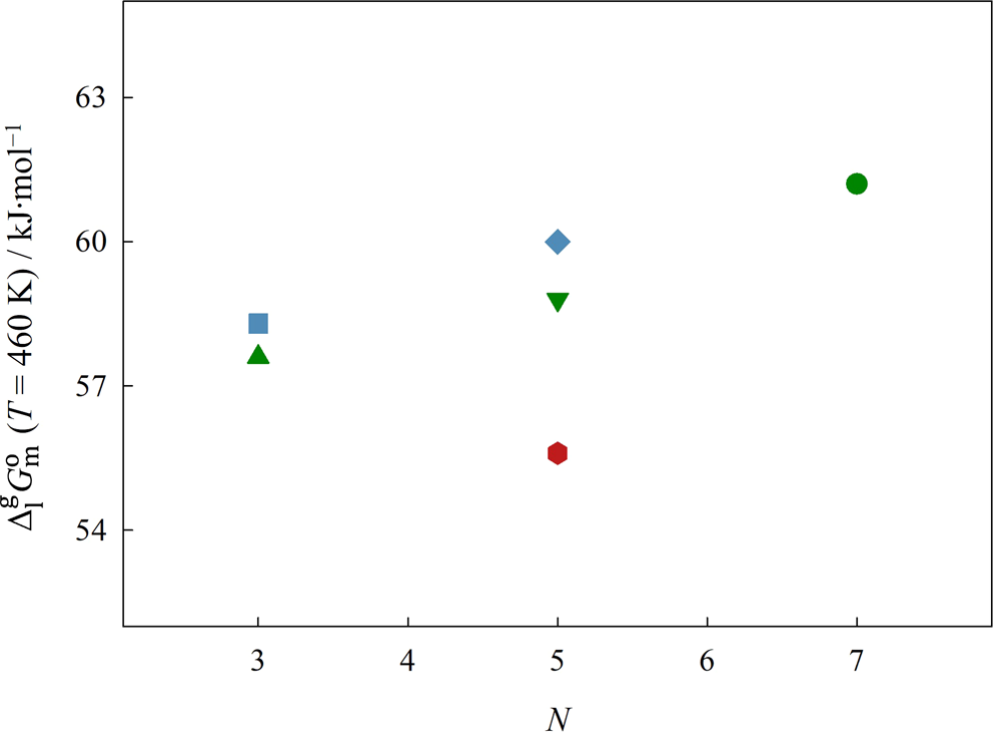
Graphical representation of the standard molar Gibbs energy of vaporization, Δ_1_^g^*G*_m_^o^, at the reference temperature of 460 K, as a function of the total number of atoms on backbone of the cation side chain. Green symbols: ▲ [(SiC)C_1_im][NTf_2_], ▼ [(SiCSiC)C_1_im][NTf_2_], ●[(SiCSiCSiC)C_1_im][NTf_2_]; blue symbols: ■ [(Np)C_1_im][NTf_2_], ◆ [(Me_4_C_5_)C_1_im][NTf_2_]; red symbols: ⬢ [(SiOSiC)C_1_im][NTf_2_]. Error bars are smaller than the symbols.

**Figure 10:**
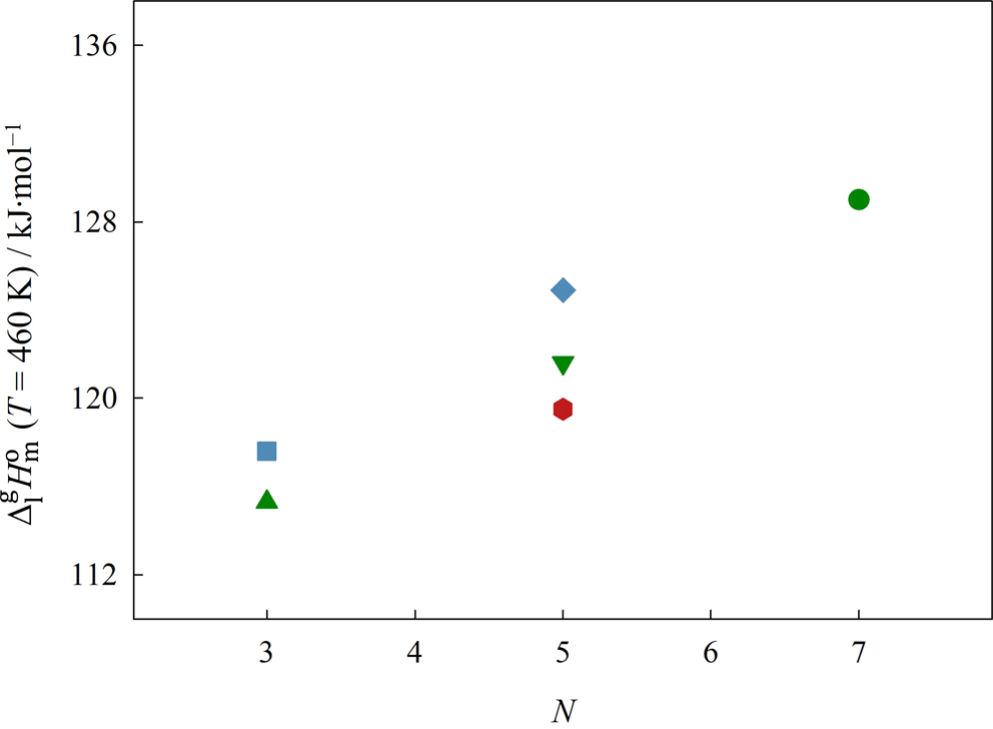
Graphical representation of the standard molar enthalpy of vaporization, Δ_1_^g^*H*_m_^o^, at the reference temperature of 460 K, as a function of the total number of atoms on the backbone of the cation side chain. Green symbols: ▲ [(SiC)C_1_im][NTf_2_], ▼ [(SiCSiC)C_1_im][NTf_2_], ●[(SiCSiCSiC)C_1_im][NTf_2_]; blue symbols: ■ [(Np)C_1_im][NTf_2_], ◆ [(Me_4_C_5_)C_1_im][NTf_2_]; red symbols: ⬢ [(SiOSiC)C_1_im][NTf_2_]. Error bars are smaller than the symbols.

**Figure 11:**
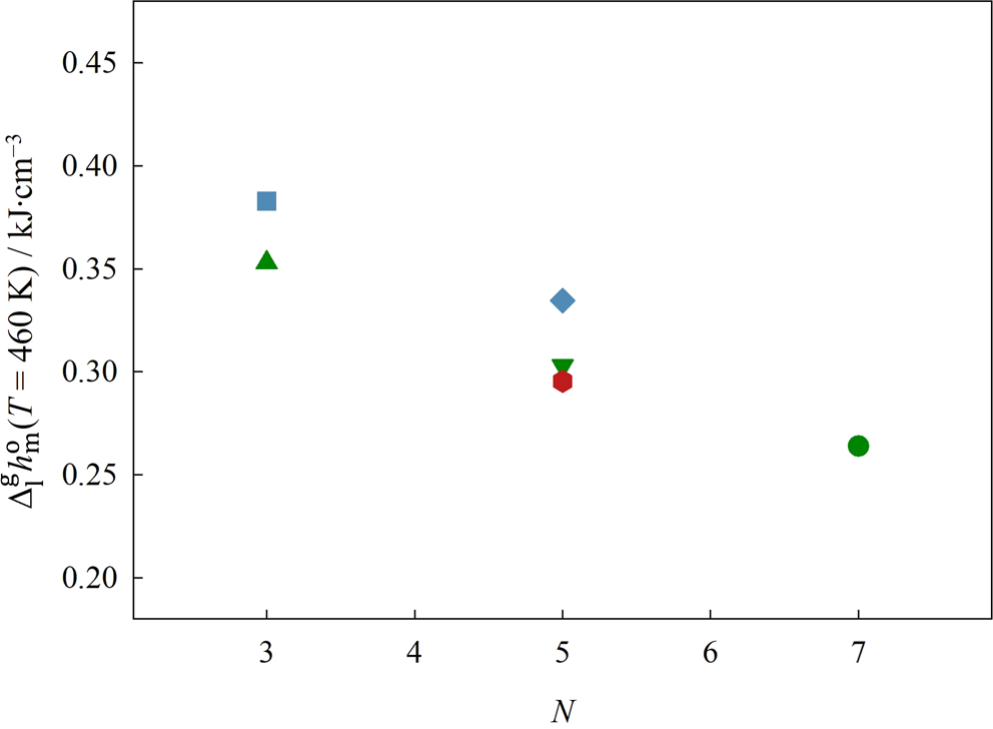
Graphical representation of the standard volumetric enthalpy of vaporization, Δ_1_^g^*h*^o^, at the reference temperature of 460 K, as a function of the total number of atoms on the backbone of the cation side chain. Green symbols: ▲ [(SiC)C_1_im][NTf_2_], ▼ [(SiCSiC)C_1_im][NTf_2_], ●[(SiCSiCSiC)C_1_im][NTf_2_]; blue symbols: ■ [(Np)C_1_im][NTf_2_], ◆ [(Me_4_C_5_)C_1_im][NTf_2_]; red symbols: ⬢ [(SiOSiC)C_1_im][NTf_2_]. Error bars are smaller than the symbols.

**Figure 12:**
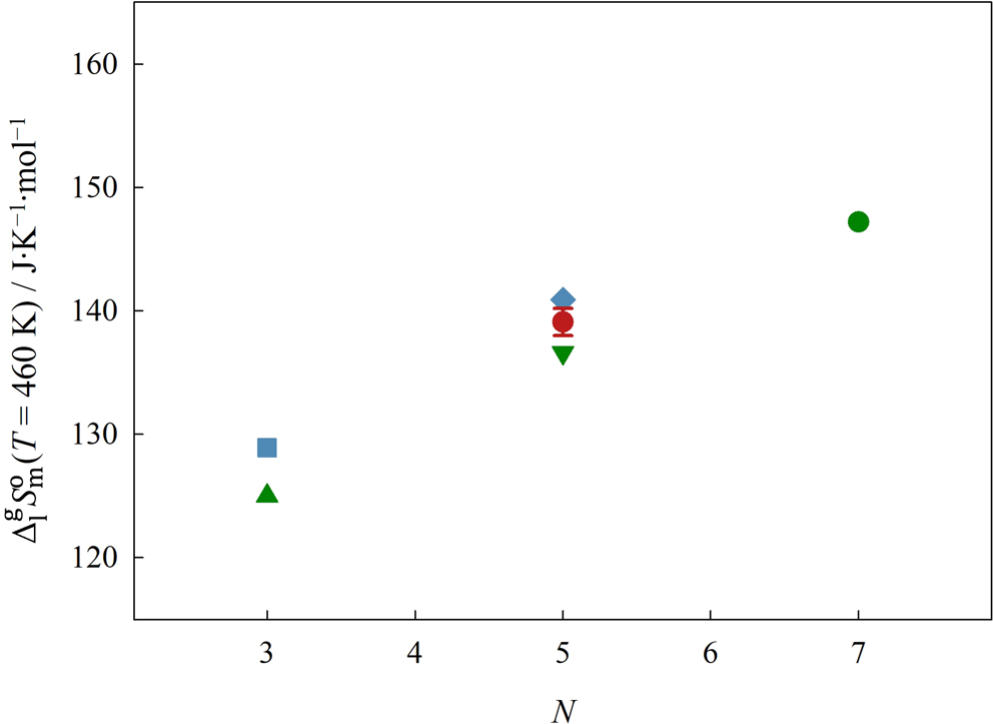
Graphical representation of the standard molar entropy of vaporization, Δ_1_^g^*S*_m_^o^, at the reference temperature of 460 K, as a function of the total number of atoms on the backbone of the cation side chain. Green symbols: ▲ [(SiC)C_1_im][NTf_2_], ▼ [(SiCSiC)C_1_im][NTf_2_], ●[(SiCSiCSiC)C_1_im][NTf_2_]; blue symbols: ■ [(Np)C_1_im][NTf_2_], ◆ [(Me_4_C_5_)C_1_im][NTf_2_]; red symbols: ⬢ [(SiOSiC)C_1_im][NTf_2_]. If not visible, error bars are smaller than the symbols.

The representation of Δ_1_^g^*G*_m_^o^, Δ_1_^g^*H*_m_^o^, and Δ_1_^g^*S*_m_^o^ at the reference temperature of 460 K, as a function of the total number of atoms in the cation chain backbone, *N*, is done in Figures 9, 10, and 12, respectively. The standard volumetric enthalpy of vaporization, Δ_1_^g^*h*^o^, at the reference temperature of 460 K is represented as a function of the number of atoms in the cation chain backbone, *N*, in Figure 11. Δ_1_^g^*h*^o^ was obtained by dividing the standard molar enthalpy of vaporization, Δ_1_^g^*H*_m_^o^, at the reference temperature of 460 K, by the molar volumes obtained by Bakis et al.^11^ (these values of molar volume correspond to the temperature of 298.15 K).

Based on our results, we verified that the replacement of a branched alkyl side chain by an analogous alkylsilane chain produces a slight decrease in the standard molar Gibbs energy of vaporization, Δ_1_^g^*G*_m_^o^. This implies that the ILs with alkylsilane chains are more volatile than their carbon-based analogs. Our results also reveal a simultaneous decrease in both the standard molar enthalpy and entropy of vaporization, Δ_1_^g^*H*_m_^o^and Δ_1_^g^*S*_m_^o^, when the quaternary carbons of the cation are replaced by silicon. Therefore, the decrease in Δ_1_^g^*G*_m_^o^ is ruled by the small reduction in the cohesive energy of the ILs, provoked by the replacement of the branched alkyl chain by an alkylsilane chain, which overcomes the decrease in the entropic contribution. The observed reduction in Δ_1_^g^*H*_m_^o^ is in agreement with the weaker cation–anion interactions found in SiILs when compared with their carbon-based analogs.^4−9^

A much more significant increase in volatility was found for the [(SiOSiC)C_1_im][NTf_2_] IL. This is a consequence of a reduced Δ_1_^g^*H*_m_^o^ together with a larger Δ_1_^g^*S*_m_^o^ when compared with its alkylsilane-based analog. The decrease in cohesive energy found for the IL with an alkylsiloxane chain can be related to the blocking of acidic H on the imidazolium ring leading to weaker cation–anion interactions and an overall weaker H-bond network.^10^ Our computational results also support this hypothesis because they suggest the occurrence of a chain-ring intramolecular interaction in [(SiOSiC)C_1_im][NTf_2_]. A previous work by Zaitsau et al.^56^ involving ether-functionalized ILs revealed that the replacement of a C3 carbon from the IL chain with oxygen produces a decrease in the IL’s Δ_1_^g^*H*_m_^o^. It is widely accepted that ether-functionalized ILs engage in intramolecular chain-ring interactions that involve the shielding of the acidic hydrogens from interacting with the anion.^32−36^ This reduction in the cation–anion interaction should also reduce the strength of the H-bonding network and is the probable cause for the decrease in Δ_1_^g^*H*_m_^o^ observed by Zaitsau et al.^56^ A similar chain-ring intramolecular interaction involving the siloxane linkage’s oxygen and an acidic ring hydrogen occurs in the alkylsiloxane chain type IL and therefore leads to a reduction of its cohesive energy interaction.

## 4 Conclusions

In this work, we evaluated the effect that introducing Si atoms and siloxane linkages on the IL cation produces on some of their thermophysical properties. We found that the increase in the length of the alkylsilane chain affects the thermal properties in an analogous way to the longer *n*-alkyl chains, with an increase in melting properties (melting point, enthalpy, and entropy of fusion), as well as an increase in the liquid cohesive energy, and a consequent decrease in volatility.

Computational results revealed that a chain-ring intramolecular interaction occurs in the [(SiOSiC)C_1_im][NTf_2_] IL, similar to those found for ether-based ILs. The introduction of a siloxane linkage seems to produce a more pronounced effect on the IL’s properties, increasing volatility and lowering the melting point. This is likely caused by the intramolecular interaction between the oxygen atom of the siloxane linkage and the acidic hydrogen atoms of the imidazolium ring, which leads to weaker interionic interactions and a consequent decrease in cohesive energy.

Good additive relationships were found for the set of studied ILs, meaning that the heat capacity of other alkylsilane and alkylsiloxane-based ILs could be easily estimated through simple group contribution models.

This work revealed that introducing Si atoms and siloxane linkages in the cation chain is a promising way to reduce the viscosity of ILs, considering that this class of ILs maintains the properties that have been regarded as desirable in ILs, such as their low vapor pressures and wide liquid range, while dramatically reducing their viscosity. This type of ionic liquid can be an alternative to the classical *n*-alkyl based ionic liquids due to their enhanced properties, which might enable new functionalities and applications. This study and conclusions can, in fact, be an inspiration for further studies and synthesis of Si-based analog ILs (e.g., pyridinium and pyrrolidinium).

## Supporting Information Available

Details of the customization and testing of the differential scanning microcalorimeter; DSC and TGA thermograms; and experimental data for KEQCM and heat capacity determination experiments (PDF)
