# Supplementary material for: Thermodynamic Study of Alkylsilane and Alkylsiloxane-Based Ionic Liquids
Source: J Phys Chem B. 2024 Apr 4;128(15):3742–54. doi: 10.1021/acs.jpcb.3c08333 (PMC11033869; doi:10.1021/acs.jpcb.3c08333)
Supplement: Supplementary file 1 — jp3c08333_si_001.pdf [file jp3c08333_si_001.pdf]

Supporting Information

Thermodynamic Study of Alkylsilane and Alkylsiloxane-based Ionic Liquids

Rodrigo M. A. Silva[1], Hadrián Montes-Campos[1], Ana I. M. C. Lobo Ferreira[1], Eduards Bakis[2], Luís M. N. B. F. Santos[1,*]

[1]CIQUP, Institute of Molecular Sciences (IMS), Department of Chemistry and Biochemistry, Faculty of Science, University of Porto, Rua do Campo Alegre, 4169-007 Porto, Portugal.

[2]Faculty of Chemistry, University of Latvia, Jelgavas 1, Riga, LV-1004, Latvia

*Corresponding author: Luís M. N. B. F. Santos

E-mail address: lbsantos@fc.up.pt

1. Customization of the SETARAM Micro DSC III S1
2. Differential scanning calorimetry S9
3. Thermogravimetric analysis S14
4. High precision drop calorimetry S24
5. Heat conduction differential scanning calorimetry S26
6. Vapor pressure measurements S26
7. Computational quantum calculations S29

References S42

1. Customization of the SETARAM Micro DSC III

This work included the customization of a commercial differential scanning microcalorimeter (SETARAM Micro DSC III). One of the main changes consisted of the assembly and installation of a temperature control module, pictured in Figure S1.

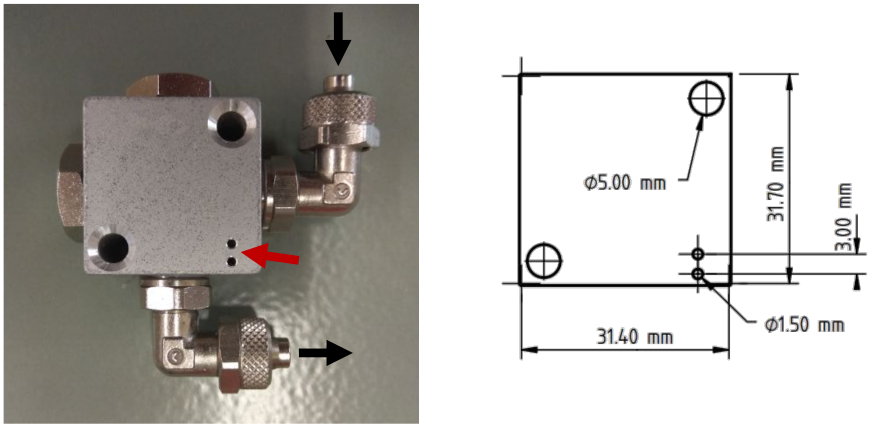

Figure S1. Local temperature control module. Black arrows indicate the flow of the thermostatic fluid. The red arrow indicates the cavities for the Pt100 temperature sensors.

This temperature control module was built from an aluminum block in which the inlet and the outlet of the thermal fluid were placed on adjacent sides. To perform the temperature readings, two Pt100 temperature sensors (connected in series) were inserted in the aluminum block, at an intermediate point between the inlet and the outlet.

Performance tests

The overall changes performed on the original apparatus allowed us to achieve very good temperature control (temperature oscillation < ±0.001 K) and obtain a calorimetric

signal with high resolution and stability (signal noise ≈ ±50 nV). Examples of temperature and signal readings performed during an isotherm at $T = 288.15$ K are shown in Figure S2.

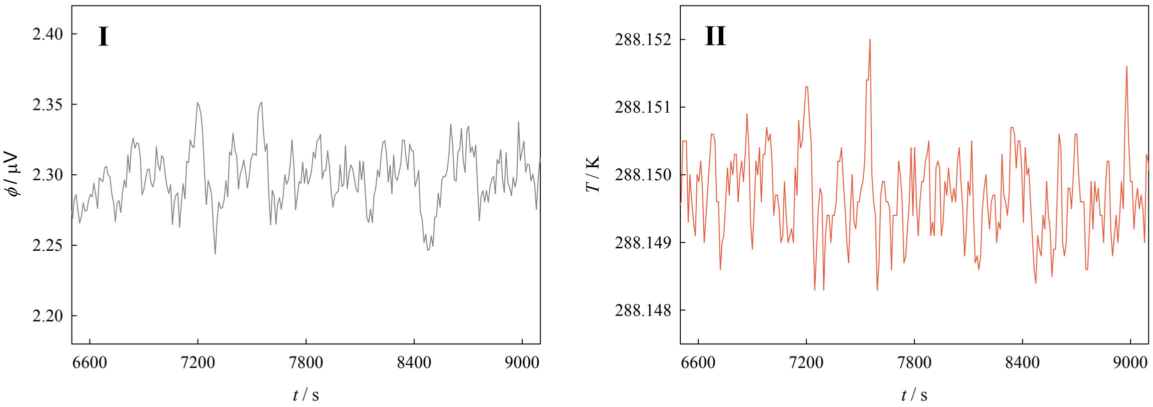

Figure S2. Calorimetric signal (I) and temperature (II) readings for an isotherm at $T = 288.15$ K.

The performance of the customized calorimeter was evaluated by measuring the heat capacity of three different test substances between 283 K and 333 K. The chosen test substances were benzoic acid (NIST SRM 39j), anthracene (Aldrich Chemical Company, >99.97 % GC analysis), and $[C_6C_1im][NTf_2]$ (Iolitec >99 %). The IL $[C_6C_1im][NTf_2]$ is the recommended IL for testing calorimetric systems which will be determining the thermophysical properties of ILs [1]. The obtained heat capacities at different experimental temperatures are presented in Table S1.

Table S1. Experimental standard molar heat capacities, $C^{o}_{p,m}$, at different temperatures for the three test substances.

T / K	$C^{o}_{p,m}$ / J·K^{-1}·mol^{-1}		
	Benzoic acid	Anthracene	[C_6C_1im][NTf_2]
283.34	358.8	421.4	482.7
293.38	362.9	426.5	488.3
303.42	366.9	431.6	493.9
313.45	371.1	436.5	499.5
323.47	375.8	442.1	505.8
333.50	380.1	447.2	511.6

Standard pressure ($p^{o} = 10^5$ Pa). The standard uncertainty of the temperature is $u(T) = 0.05$ K, and the combined expanded uncertainty (0.95 level of confidence, $k \approx 2$) of the heat capacity is U_c ($C^{o}_{p,m}$) = 0.075·$C^{o}_{p,m}$.

These heat capacity results were fitted to a linear equation,

$$C^{o}_{p,m}(T) = a + b \cdot (T / \mathrm{K}) \qquad \text{(S1)}$$

Table S2 contains the a and b parameters obtained for the different test substances.

Table S1. Parameters obtained from the linear fitting of the heat capacity, as a function of temperature for the different test substances.

Test substance	a / J·K^{-1}·mol^{-1}	b / J·K^{-2}·mol^{-1}	s_r (%)
Benzoic acid	238.0 ± 1.8	0.426 ± 0.006	0.08
Anthracene	275.5 ± 1.3	0.515 ± 0.004	0.05
[C_6C_1im][NTf_2]	453.8 ± 2.7	0.783 ± 0.008	0.02

$s_r = \left(\frac{\left(C^{o}_{p,m} - C^{o}_{p,m,\mathrm{fit}}\right) / C^{o}_{p,m,\mathrm{fit}}}{n-m} \right) \times 100$, in which n is the number of fitted data points and m is the number of independent adjustable parameters (here $m = 2$).

The heat capacity at T = 298.15 K obtained for each of the test substances are presented in Table S3, along with selected results found in the literature. The relative deviation plots, as a function of temperature, between heat capacity data found in the literature and the linear fit obtained in this work (Table S2) are presented in Figures S3 to S5.

Table S3. Standard molar isobaric heat capacity ($C^{o}_{p,m}$) at T = 298.15 K for the test substances.

Test substance	$C^{o}_{p,m}$ (T = 298.15 K) / $J \cdot K^{-1} \cdot mol^{-1}$
Benzoic acid	146.8 ± 1.1 (this work)
	146.81 ± 0.07 [2]
	147.166 ± 0.294 [3]
	146.778 ± 0.073 [4]
Anthracene	210.3 ± 1.6 (this work)
	210.5 ± 0.2 [5]
	211.7 ± 2.5 [6]
$[C_6C_1im][NTf_2]$	628.9 ± 4.7 (this work)
	629.4 ± 1.2 [7]
	629.1 ± 2.2 [8]
	629.2 ± 2.5 [9]

Standard pressure (p^{o} = 10^5 Pa). The combined expanded uncertainty (0.95 level of confidence, $k \approx 2$,) of the heat capacity is U_c ($C^{o}_{p,m}$) = 0.075· $C^{o}_{p,m}$.

The obtained heat capacities, for all the test substances, are in very good agreement with results found in the literature, which were mostly obtained with high-precision techniques such as adiabatic calorimetry or drop calorimetry. For benzoic acid, the heat capacity determined in this work presented smaller dependence on temperature when compared with the heat capacity reported by Furukawa *et al.* [2] and by Moriya *et al.* [3]. Nonetheless, the

deviations between our results and the ones published by those authors are smaller than 0.3 % and 0.6 %, respectively, across the whole temperature range.

The heat capacities obtained by Goursot *et al*. [5] for anthracene present a larger heat capacity dependence on temperature than the ones we obtained in the performance tests. Although, those reported by Radomska *et al*. [6] show a smaller heat capacity dependence on temperature when compared to our results. In both cases, the deviations between our results and the ones determined by those authors are smaller than 0.8 %.

Finally, for $[C_6C_1im][NTf_2]$, the results obtained in the performance tests are in excellent agreement with those previously reported by our group [7], the ones suggested by Paredes *et al*. [8], and those published by Blokhin *et al*. [9]. The dependence of heat capacity on temperature suggested by Paredes *et al*. for $[C_6C_1im][NTf_2]$ is nearly identical to the one we have obtained in the performance tests, as the deviations across the whole temperature range are smaller than 0.1%.

Through the performance tests, it was possible to verify that the refurbished version of the HC-DSC is able to reproduce data from the literature that was obtained with high-precision techniques. Therefore, we can state that the refurbished version of the HC-DSC is a high-precision technique for the determination of heat capacities of solids and liquids, in the temperature range from 283 K to 333 K.

Deviation plots

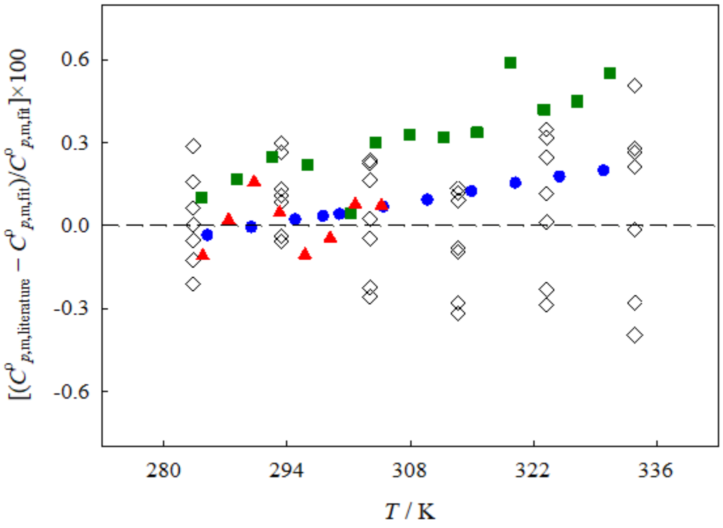

Figure S3. Relative deviation of literature data to our fit, as a function of temperature, for crystalline benzoic acid. ●: Furukawa *et al*. [2], ■: Moriya *et al*. [3], ▲: Sorai *et al*. [4], ◇: this work.

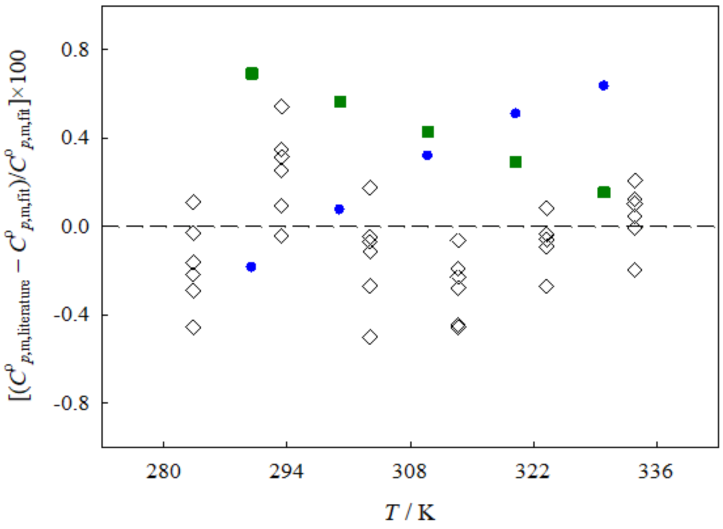

Figure S4. Relative deviation of literature data to our fit, as a function of temperature, for crystalline anthracene. ●: Goursot *et al*. [5], ■: Radomska *et al*. [6], ◇: this work.

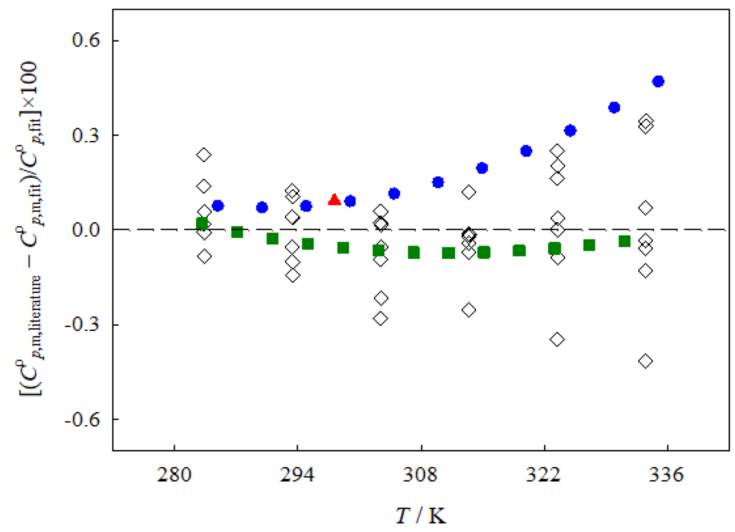

Figure S5. Relative deviation of literature data to our fit, as a function of temperature, for liquid $[C_6C_1im][NTf_2]$. ●: Blokhin *et al.* [7], ■: Paredes *et al.* [8], ▲: Rocha *et al.* [9],◇: this work.

Evaluation of the calorimeter's time constant

Another of the evaluated parameters was the time constant of the calorimeter at different temperatures. The time constant is defined as the ratio between the total heat capacity of the group that comprises the calorimetric cell (and its content), the cell holder and half of the heat flow sensors (C), and the thermal conductivity of the heat flow sensors (G), as shown in Equation S2.

$$\tau = \frac{C}{G} \tag{S2}$$

The time constant determination was done by fitting the calorimetric signal's return to the baseline to an exponential decay:

$$\phi = A \cdot e^{-\frac{t}{\tau}} \tag{S3}$$

$$\ln(\phi) = -\frac{1}{\tau} \cdot t + \ln(A) \tag{S4}$$

in which ϕ is the calorimetric signal, A is a pre-exponential factor, t is time and τ is the time constant. The representation of the calculated time constants as a function of temperature is done in Table S4 and Figure S6.

Table S4. Time constant at the different experimental temperatures.

T / K	τ / s
298.40	41.9 ± 0.2
308.43	42.6 ± 0.2
318.46	43.2 ± 0.2
328.49	44.2 ± 0.2
338.51	44.9 ± 0.2

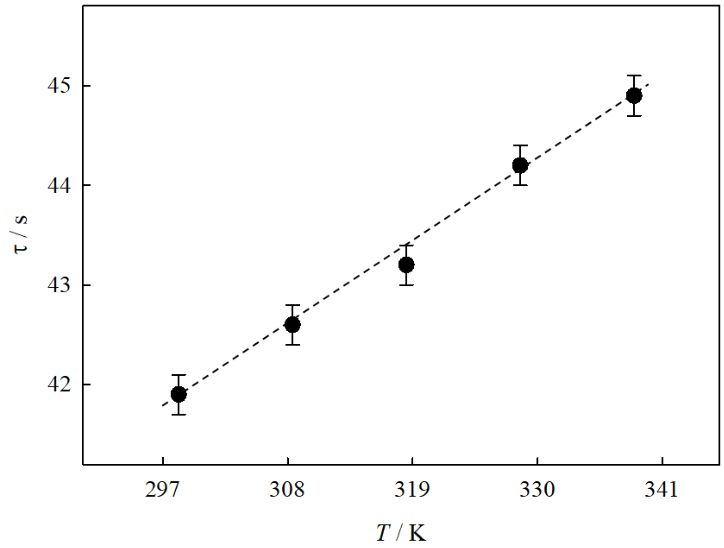

Figure S6. Graphical representation of the calculated time constants (τ) for different temperatures (T). The dashed line is a guide to the eye.

Time constant, τ, becomes higher as the temperature is increased. This observation should be associated with an increase in the total heat capacity (C in Equation S2) and the decrease of thermal conductivity.

2. Differential scanning calorimetry

The DSC samples were weighted in a Mettler Toledo UMT2 micro balance, with a resolution of 0.0001 mg and estimated uncertainty of $\pm$ 0.0005 mg. The thermograms for the various studied ILs are presented from Figure S7 to Figure S12.

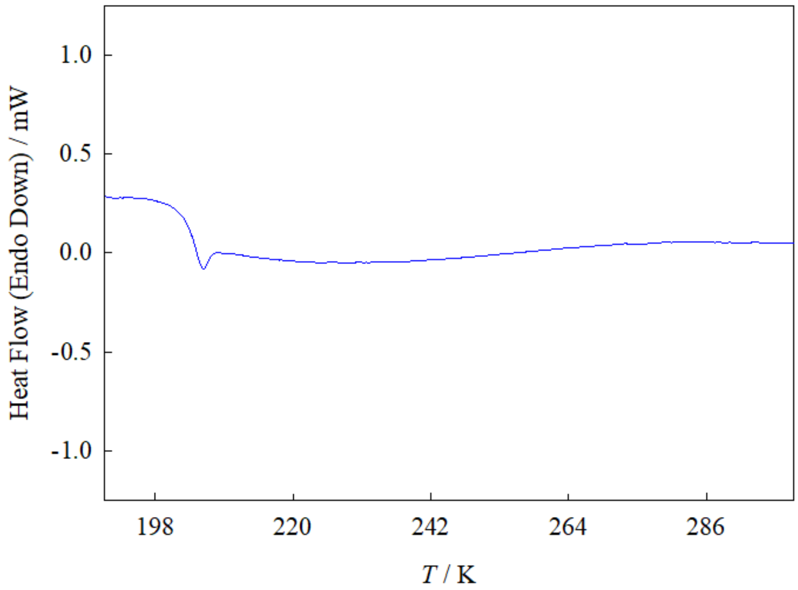

Figure S7. Thermogram obtained for the heating (solid blue line) of [(SiC)C_1im][NTf_2] (endothermic peak down). Heating rate $\beta = 5\ K\cdot min^{-1}$, sample mass $m_{sample} = 7.7284$ mg.

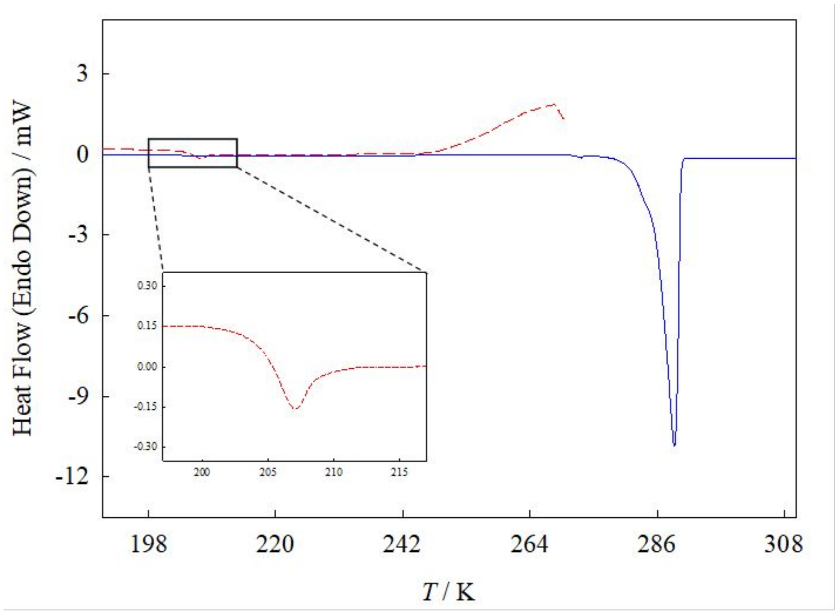

Figure S8. Thermograms obtained for the first (dashed red line) and second (solid blue line) heating of [(SiCSiC)C_1im][NTf_2] (endothermic peak down). Heating rate β = 5 K·min^{-1}, sample mass m_{sample} = 10.3505 mg.

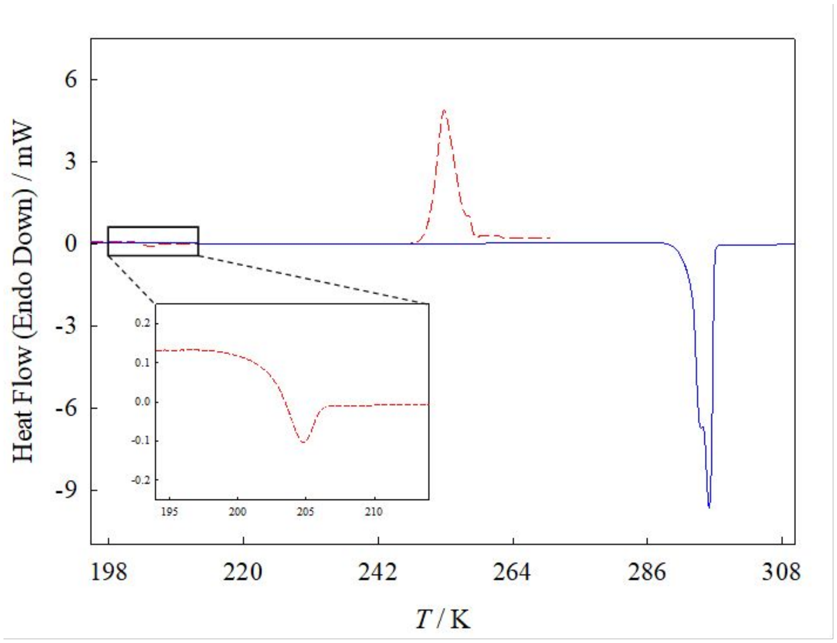

Figure S9. Thermograms obtained for the first (dashed red line) and second (solid blue line) heating of [(SiCSiCSiC)C_1im][NTf_2] (endothermic peak down). Heating rate β = 5 K·min^{-1}, sample mass m_{sample} = 5.5220 mg.

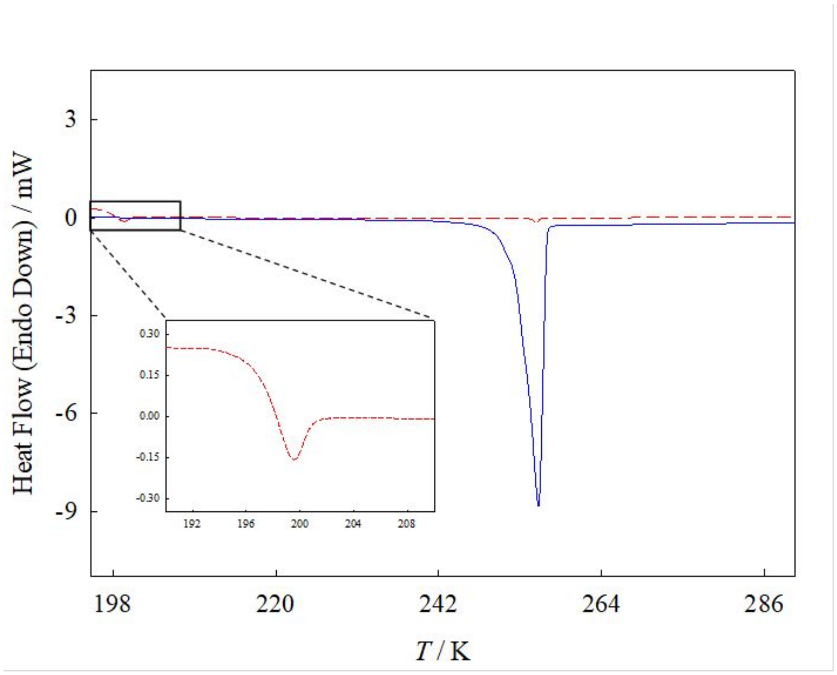

Figure S10. Thermograms obtained for the first (dashed red line) and second (solid blue line) heating of [(SiOSiC)C_1im][NTf_2] (endothermic peak down). Heating rate β = 5 K·min^{-1}, sample mass m_{sample} = 11.0156 mg.

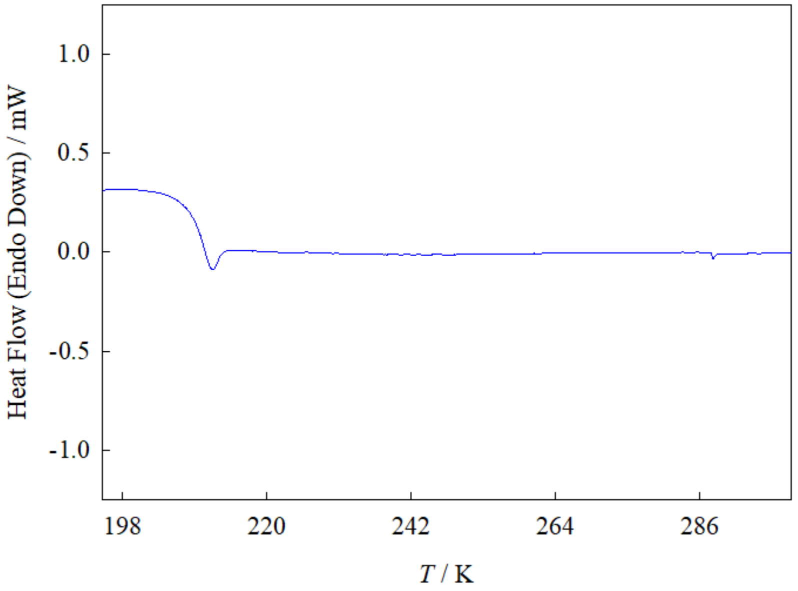

Figure S11. Thermogram obtained for the heating (solid blue line) of [(Np)C_1im][NTf_2] (endothermic peak down). Heating rate β = 5 K·min^{-1}, sample mass m_{sample} = 9.9842 mg.

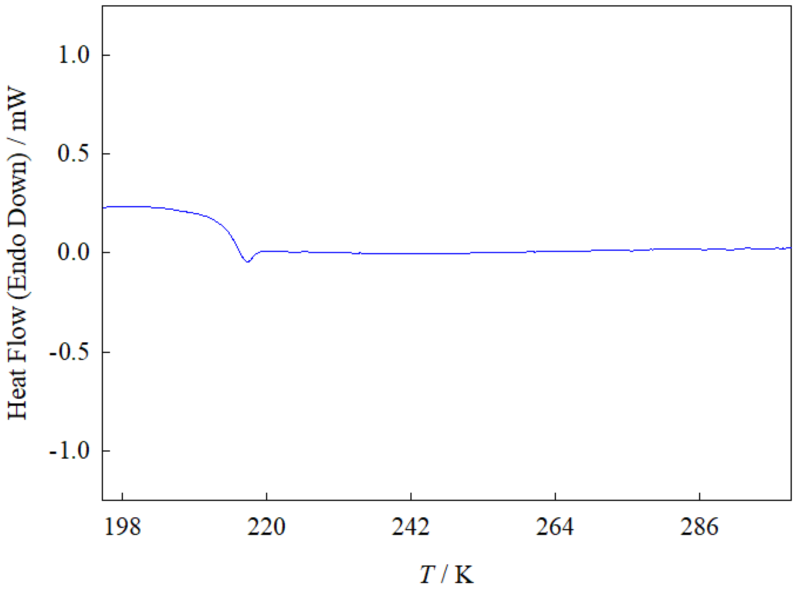

Figure S12. Thermogram obtained for the heating (solid blue line) of [$(Me_4C_5)C_1$im][NTf_2] (endothermic peak down). Heating rate β = 5 K·min^{-1}, sample mass m_{sample} = 6.9026 mg.

3. Thermogravimetric analysis

The thermograms obtained for the various studied ILs by TGA are presented from Figure S13 to Figure S18.

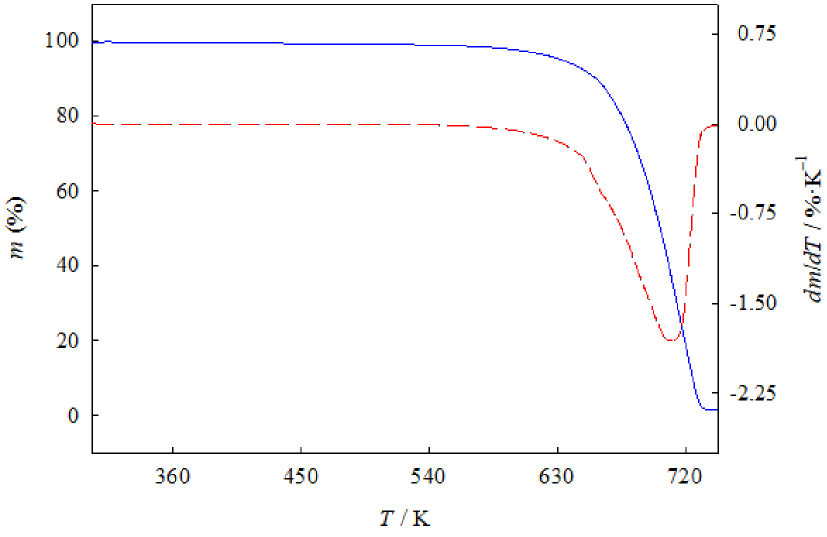

Figure S13. Mass loss (solid blue line) and mass loss derivative (dashed red line) for $[(SiC)C_1im][NTf_2]$. Heating rate $\beta = 5$ K·min^{-1}, sample mass $m_{sample} = 9.862$ mg.

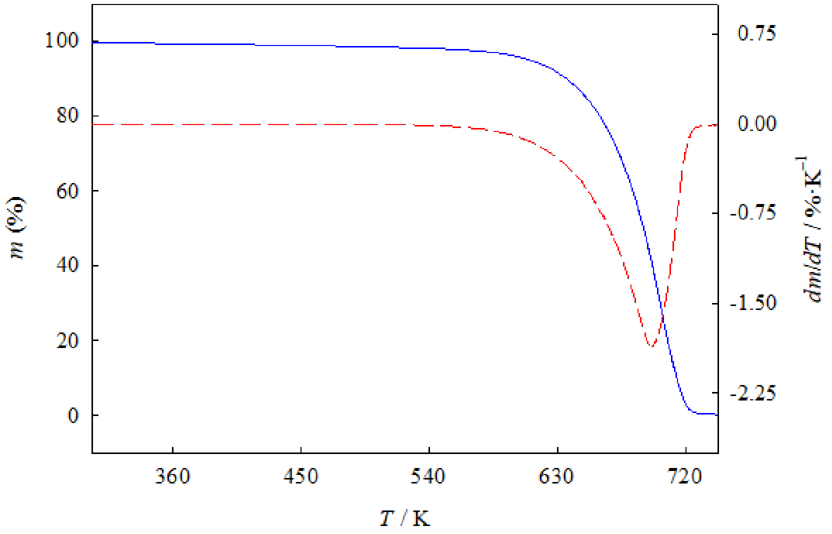

Figure S14. Mass loss (solid blue line) and mass loss derivative (dashed red line) for $[(SiCSiC)C_1im][NTf_2]$. Heating rate $\beta = 5$ K·min^{-1}, sample mass $m_{sample} = 8.267$ mg.

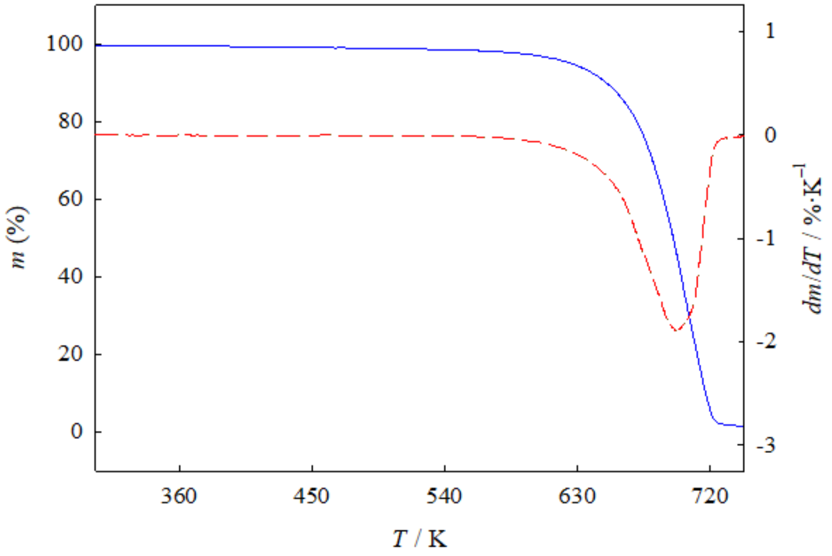

Figure S15. Mass loss (solid blue line) and mass loss derivative (dashed red line) for $[(SiCSiCSiC)C_1im][NTf_2]$. Heating rate $\beta = 5$ K·min^{-1}, sample mass $m_{sample} = 8.137$ mg.

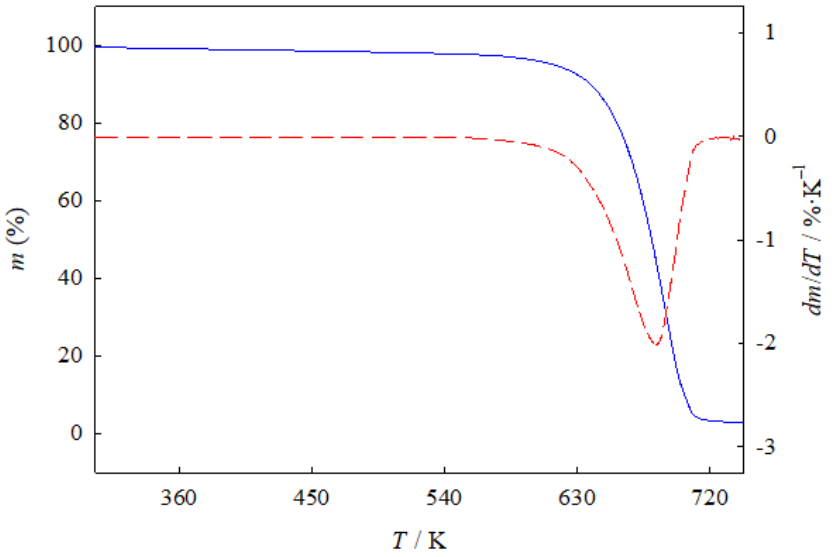

Figure S16. Mass loss (solid blue line) and mass loss derivative (dashed red line) for [(Np)C_1im][NTf_2]. Heating rate β = 5 K·min^{-1}, sample mass m_{sample} = 14.030 mg.

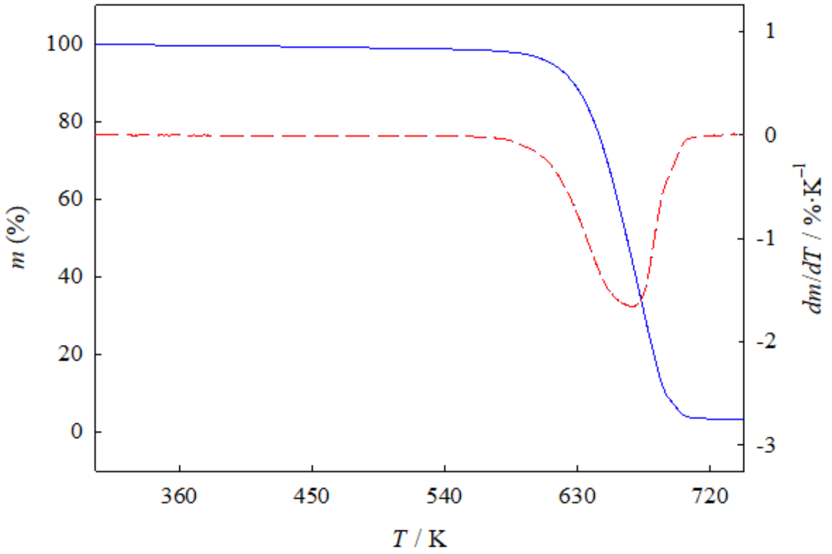

Figure S17. Mass loss (solid blue line) and mass loss derivative (dashed red line) for [(Me_4C_5)C_1im][NTf_2]. Heating rate β = 5 K·min^{-1}, sample mass m_{sample} = 11.194 mg.

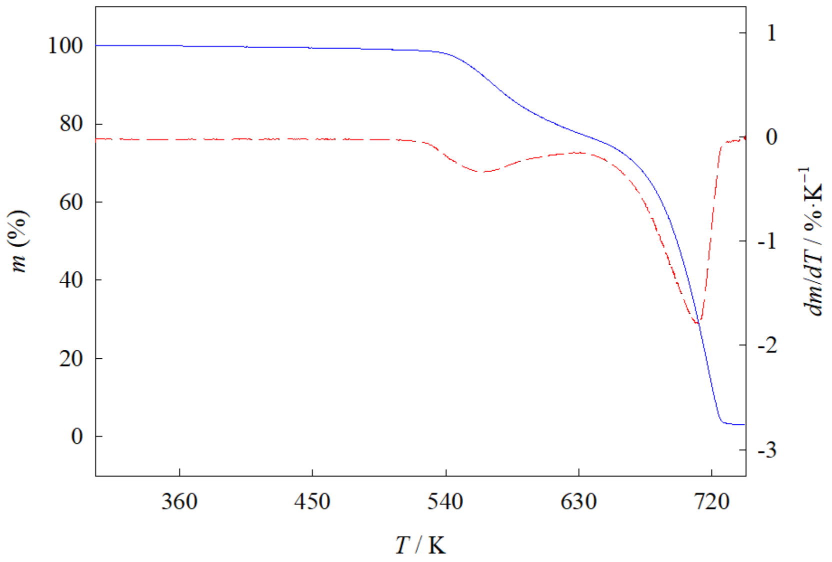

Figure S18. Mass loss (solid blue line) and mass loss derivative (dashed red line) for $[(SiOSiC)C_1im][NTf_2]$. Heating rate $\beta = 5$ K·min^{-1}, sample mass $m_{sample} = 5.795$ mg.

The overall comparison of the TGA thermograms obtained for the different ILs is done in Figure S19 (superimposition of the TGA thermograms). The onset decomposition temperatures, obtained at four different scanning rates ($\beta = 0.8, 2, 5, 10$ K·min^{-1}) for the different studied ILs are presented in Table S5.

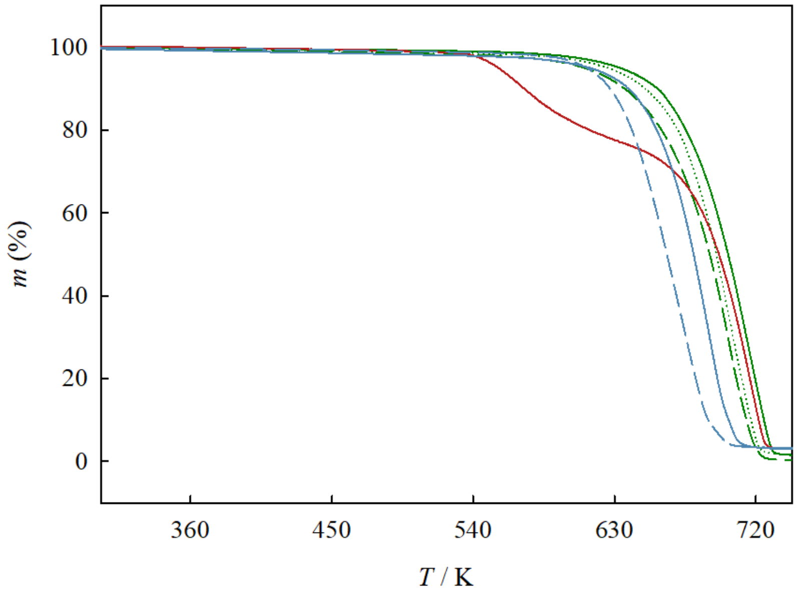

Figure S19. Themograms of the TGA experiments performed at β = 5 K·min^{-1}. Green lines: [(SiC)C_1im][NTf_2] (solid), [(SiCSiC)C_1im][NTf_2] (dashed), [(SiCSiCSiC)C_1im][NTf_2] (dotted); Blue lines: [(Np)C_1im][NTf_2] (solid), [(Me_4C_5)C_1im][NTf_2] (dashed); Red line: [(SiOSiC)C_1im][NTf_2].

Table S5. Decomposition temperature, T_d, for the studied ionic liquids as a function of the scanning rate, β.

	T_d / K			
β / K·min^{-1}	0.8	2	5	10
[(SiC)C_1im][NTf_2]	629.0	648.8	680.8	695.4
[(SiCSiC)C_1im][NTf_2]	625.2	651.1	669.1	681.6
[(SiCSiCSiC)C_1im][NTf_2]	631.4	649.5	670.5	685.7
[(Np)C_1im][NTf_2]	615.9	636.3	657.6	668.1
[(Me_4C_5)C_1im][NTf_2]	608.5	622.0	634.1	643.0
[(SiOSiC)C_1im][NTf_2]	519.1	535.8	546.0	566.4

T_d was fitted as a function of $\beta^{1/3}$ according to the following equation,

$$T_d(\beta^{1/3}) = a + b \cdot \left(\beta / \mathrm{K} \cdot \mathrm{min}^{-1}\right)^{1/3} \tag{S5}$$

The plots of T_d as a function of $\beta^{1/3}$ for the studied ILs is done in Figures S20 to S25. We chose to fit T_d as a function of $\beta^{1/3}$ since this model provided the best linear extrapolation. The a and b parameters obtained from the fitting are presented in Table S6.

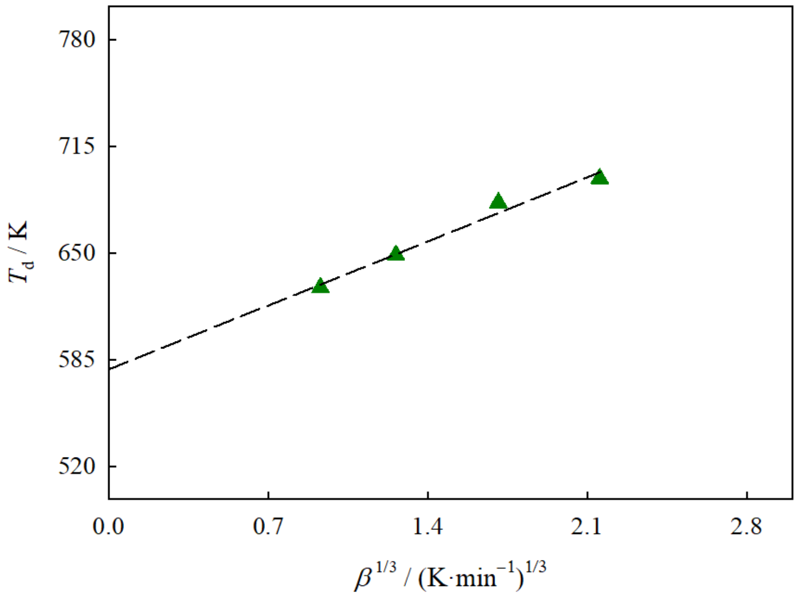

Figure S20. Plot of T_d as a function of $\beta^{1/3}$ for [(SiC)C_1im][NTf_2]. The dashed line represents the linear fit of the experimental points (▲).

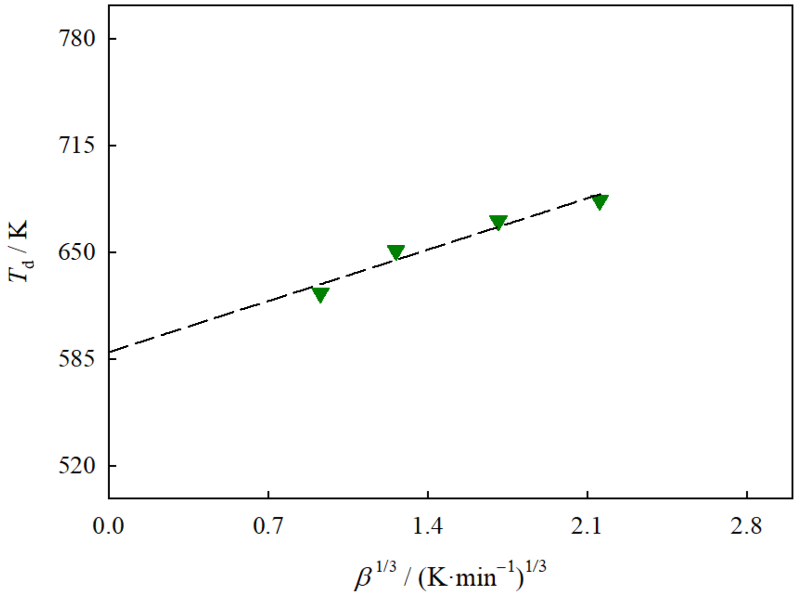

Figure S21. Plot of T_d as a function of $\beta^{1/3}$ for [(SiCSiC)C_1im][NTf_2]. The dashed line represents the linear fit of the experimental points (▼).

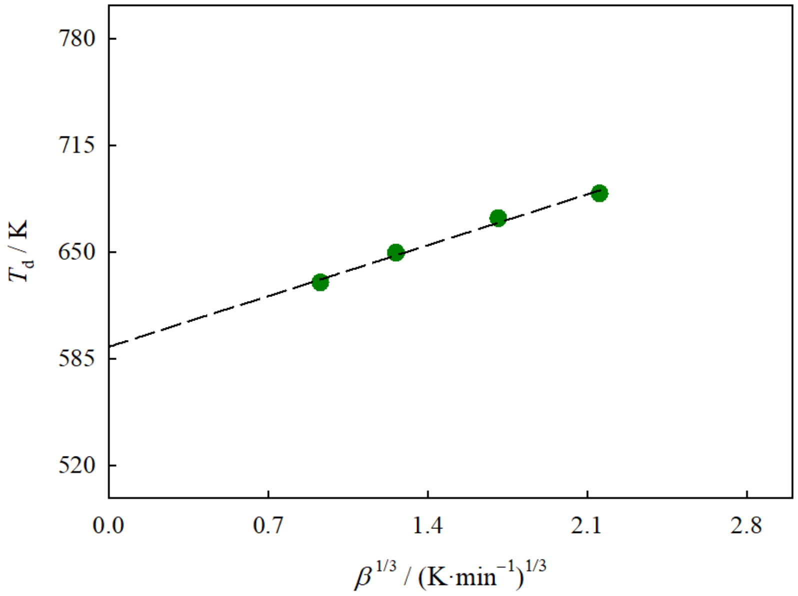

Figure S22. Plot of T_d as a function of $\beta^{1/3}$ for [(SiCSiCSiC)C_1im][NTf_2]. The dashed line represents the linear fit of the experimental points (●).

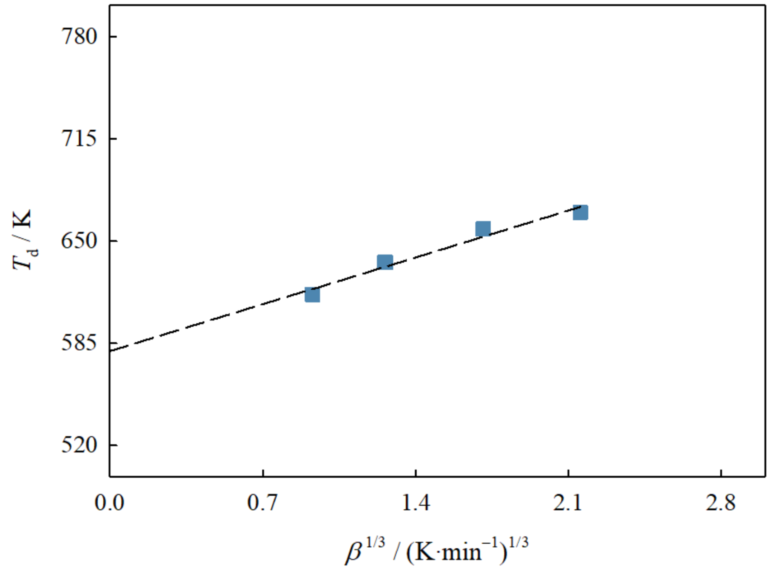

Figure S23. Plot of T_d as a function of $\beta^{1/3}$ for [(Np)C_1im][NTf_2]. The dashed line represents the linear fit of the experimental points (■).

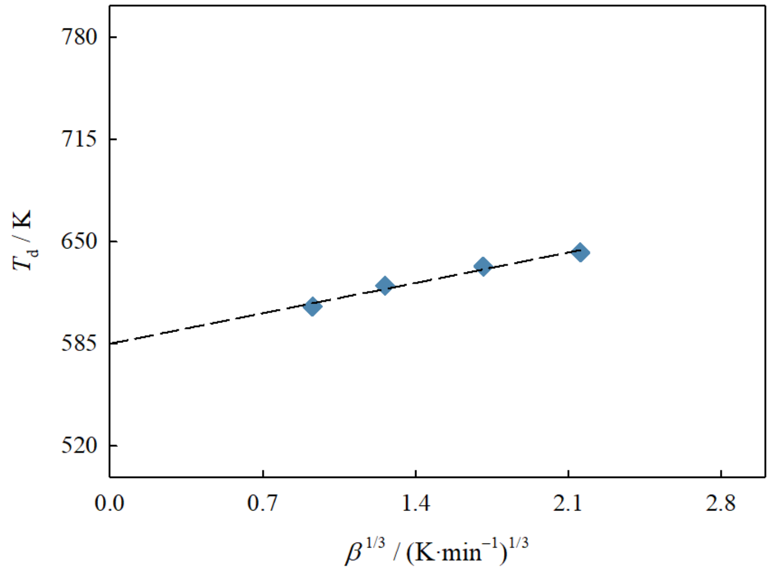

Figure S24. Plot of T_d as a function of $\beta^{1/3}$ for [(Me_4C_5)C_1im][NTf_2]. The dashed line represents the linear fit of the experimental points (◆).

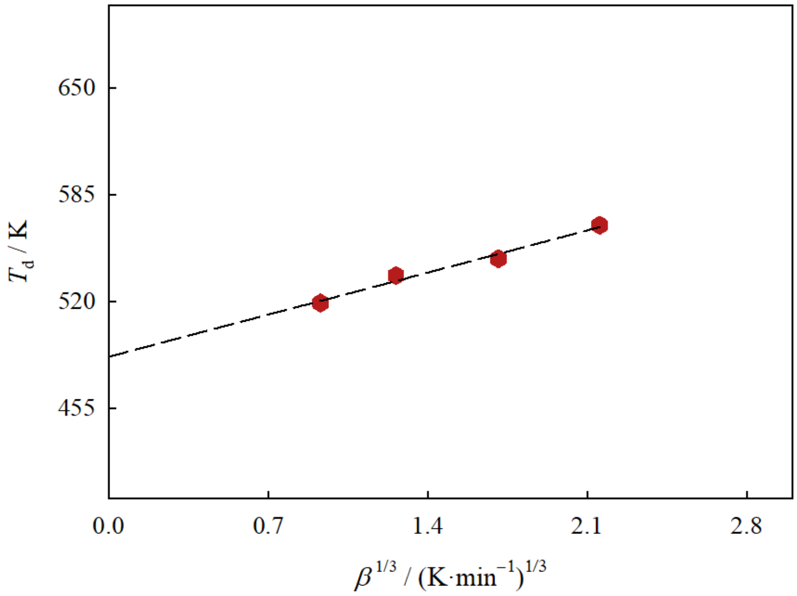

Figure S25. Plot of T_d as a function of $\beta^{1/3}$ for [(SiOSiC)C_1im][NTf_2]. The dashed line represents the linear fit of the experimental points (□).

Table S6. Parameters obtained from the fitting of T_d as a function of $\beta^{1/3}$.

Ionic Liquid	a / K	b / (K^2·min)$^{1/3}$	R^2
[(SiC)C_1im][NTf_2]	579.0 ± 9.3	55.77 ± 5.86	0.9784
[(SiCSiC)C_1im][NTf_2]	589.1 ± 11.4	44.68 ± 7.19	0.9507
[(SiCSiCSiC)C_1im][NTf_2]	592.2 ± 4.9	44.31 ± 3.07	0.9905
[(Np)C_1im][NTf_2]	579.9 ± 9.1	42.65 ± 5.74	0.9651
[(Me_4C_5)C_1im][NTf_2]	584.9 ± 4.7	27.73 ± 2.97	0.9775
[(SiOSiC)C_1im][NTf_2]	486.3 ± 5.7	36.67 ± 3.64	0.9807

4. High precision drop calorimetry

Table S7 contains details about the high-precision drop calorimetry experiments. The heat capacities obtained for each individual experiment are presented, along with experimental details.

Table S7. Standard molar heat capacity $C^{\circ}_{p,\mathrm{m}}$ results at T = 298.15 K for the different ionic liquids. The calibration constant used to calculate the $C^{\circ}_{p,\mathrm{m}}$ was derived from the sapphire (NBS, SRM 720, α-Al_2O_3) calibration.

Ionic Liquid	Exp	m/g	N_{drop}	T_{fur}/K	T_{cal}/K	T/K	$C^{\circ}_{p,\mathrm{m}}$ /J·K^{-1}·mol^{-1}
[(SiC)C_1im][NTf_2]	I	0.52568	11	303.21	293.18	298.20	625.1 ± 2.1
(l)	II	0.59035	46	303.25	293.18	298.22	626.8 ± 1.8
	III	0.57113	32	303.12	293.18	298.15	624.0 ± 1.9
	IV	0.58346	10	303.23	293.18	298.20	625.0 ± 1.6
[(SiCSiC)C_1im]	I	0.44508	47	303.19	293.18	298.19	767.2 ± 2.4
[NTf_2] (l)	II	0.41472	38	303.17	293.18	298.18	768.3 ± 2.4
	III	0.53336	33	303.24	293.18	298.21	767.6 ± 2.3
[(SiCSiCSiC)C_1im]	I	0.46162	37	303.21	293.18	298.20	908.5 ± 2.2
[NTf_2] (l)	II	0.48882	10	303.19	293.18	298.19	908.8 ± 2.5
	III	0.46066	24	303.20	293.18	298.19	909.5 ± 2.0
[(Np)C_1im]	I	0.62760	33	303.21	293.18	298.20	594.7 ± 1.7
[NTf_2] (l)	II	0.57411	8	303.21	293.18	298.20	595.1 ± 1.8
	III	0.60050	11	303.20	293.19	298.19	596.2 ± 1.7
[(Me_4C_5)C_1im]	I	0.50108	18	303.25	293.18	298.21	708.4 ± 1.8
[NTf_2] (l)	II	0.50244	35	303.26	293.18	298.22	709.2 ± 1.7
	III	0.48307	19	303.22	293.18	298.20	707.8 ± 1.6
[(SiOSiC)C_1im]	I	0.50099	50	303.20	293.18	298.19	745.5 ± 1.9
[NTf_2] (l)	II	0.45875	4	303.15	293.18	298.17	744.0 ± 2.4
	III	0.45362	11	303.20	293.18	298.19	744.3 ± 2.7

N_{drop} = number of drop experiments; T_{fur} = average temperature of the furnace; T_{cal} = average temperature of the calorimeter; the uncertainty reported is twice the standard deviation of the mean and the calibration uncertainty is included.

5. Heat conduction differential scanning calorimetry

The experimental heat capacity data obtained for each run with the heat conduction type differential scanning microcalorimetry (HC-DSC) are presented in Table S8. For the HC-DSC measurements, ΔT_{step} = 10 K, β = 0.3 K·min^{-1}, $t_{\mathrm{isothermal}}$ = 3600 s. The HC-DSC samples were weighted in a Mettler Toledo AT201 analytical balance, with a resolution of 0.01 mg (estimated uncertainty ± 0.02 mg).

Table S8. Experimental standard molar heat capacities, $C^{\mathrm{o}}_{p,\mathrm{m}}$, at the different experimental temperatures for the liquid phase of the studied ionic liquids

	$C^{\mathrm{o}}_{p,\mathrm{m}}$ / J·K^{-1}·mol^{-1}					
T / K	283.34	293.38	303.42	313.45	323.47	333.50
[(SiC)C_1im][NTf_2]	616.9	622.7	628.3	633.4	639.9	645.9
[(SiCSiC)C_1im][NTf_2]	759.4	767.5	774.8	782.1	790.7	798.2
[(SiCSiCSiC)C_1im][NTf_2]	893.8	904.1	913.5	922.8	933.5	943.2
[(Np)C_1im][NTf_2]	586.2	592.8	598.9	605.3	612.3	618.6
[(Me_4C_5)C_1im][NTf_2]	693.3	702.6	712.3	721.0	731.1	740.9
[(SiOSiC)C_1im][NTf_2]	736.0	743.2	749.4	755.7	763.1	770.1

The standard uncertainty of the temperature is $u(T)$ = 0.05 K, and the combined expanded uncertainty of the heat capacity is $U_{\mathrm{c}}(C^{\mathrm{o}}_{p,\mathrm{m}}) = 0.075 \cdot C^{\mathrm{o}}_{p,\mathrm{m}}$ ($k \approx 2$, 0.95 level of confidence).

6. Vapor pressure measurements

The vapor pressures obtained at the different experimental temperatures in the KEQCM experiments are presented in Table S9. The parameters obtained from the fitting of the experimental vapor pressures to the Clarke and Glew equation are presented in Table S10.

Table S9. Vapor pressures, p, determined at the different experimental temperatures, T.

T / K	p / Pa	Δp / Pa	T / K	p / Pa	Δp / Pa	T / K	p / Pa	Δp / Pa
				[(SiC)C_1im][NTf_2]				
448.19	0.0132	0.0001	463.20	0.0357	−0.0001	478.19	0.0910	0.0001
453.20	0.0183	−0.0002	468.19	0.0495	0.0002	483.18	0.1222	−0.0002
458.20	0.0259	0.0001	473.19	0.0667	−0.0005	488.18	0.1634	0.0008
				[(SiCSiC)C_1im][NTf_2]				
453.19	0.0131	0.0001	468.18	0.0364	−0.0001	483.17	0.0949	−0.0001
458.19	0.0185	0.0000	473.17	0.0507	0.0000	488.17	0.1288	0.0004
463.19	0.0260	−0.0001	478.17	0.0696	−0.0001	493.16	0.1734	0.0011
				[(SiCSiCSiC)C_1im][NTf_2]				
463.18	0.0140	0.0001	478.17	0.0397	0.0001	493.16	0.1036	0.0002
468.17	0.0199	0.0000	483.17	0.0550	0.0000	493.16	0.1403	0.0002
473.17	0.0282	0.0000	488.16	0.0757	0.0000	503.13	0.1885	0.0004
				[(Np)C_1im][NTf_2]				
448.18	0.0106	0.0000	463.17	0.0295	0.0000	478.16	0.0763	0.0002
453.18	0.0151	0.0001	468.17	0.0406	−0.0002	483.16	0.1030	0.0003
458.17	0.0211	0.0000	473.17	0.0561	0.0001	488.16	0.1383	0.0007
				[(Me_4C_5)C_1im][NTf_2]				
458.14	0.0135	0.0000	473.13	0.0378	−0.0001	488.11	0.0983	0.0001
463.13	0.0192	0.0000	478.12	0.0525	0.0000	493.10	0.1330	0.0004
468.13	0.0271	0.0000	483.11	0.0719	−0.0001	498.10	0.1782	0.0004
				[(SiOSiC)C_1im][NTf_2]				
443.15	0.0147	−0.0001	458.14	0.0434	0.0002	473.12	0.1162	0.0000
448.15	0.0215	0.0001	463.13	0.0609	0.0003	478.10	0.1581	−0.0006
453.14	0.0306	0.0001	468.13	0.0845	0.0002			

$\Delta p = p - p_{calc}$, where p_{calc} is calculated from the Clarke and Glew equation (Equation (4)) and the parameters from Table S10. Standard uncertainties, u, are $u(T) = 0.02$ K and $u(p) = (0.001 + 0.015 \cdot p)$ Pa, at the 0.95 confidence level (k ≈ 2).

Table S10. Parameters obtained from the fitting of the Clarke and Glew equation, mean experimental temperature, $\langle T \rangle$, and pressure at the mean experimental temperature, $p(\langle T \rangle)$, for each of the studied ionic liquids.

Ionic Liquid	$\Delta_l^g G_m^o$ / J·mol^{-1}	$\Delta_l^g H_m^o$ / J·mol^{-1}	R^2	$\langle T \rangle$ / K	$p(\langle T \rangle)$ / Pa
[(SiC)C_1im][NTf_2]	56538 ± 8	114639 ± 293	0.99995	468.19	0.0492
[(SiCSiC)C_1im][NTf_2]	57028 ± 5	120187 ± 194	0.99998	473.18	0.0507
[(SiCSiCSiC)C_1im][NTf_2]	57907 ± 1	126099 ± 53	0.99999	483.16	0.0549
[(Np)C_1im][NTf_2]	57269 ± 4	116769 ± 140	0.99999	468.17	0.0408
[(Me_4C_5)C_1im][NTf_2]	57486 ± 2	122474 ± 65	0.99999	478.12	0.0525
[(SiOSiC)C_1im][NTf_2]	55473 ± 8	119487 ± 305	0.99996	460.63	0.0512

Standard uncertainties, u, are $u(T)$ = 0.02 K and $u(p)$ = (0.001 + 0.015 · p) Pa, at the 0.95 confidence level ($k \approx 2$).

In order to obtain the derivative of $\Delta_l^g C_{p,m}^o(T)$, the function of $\Delta_l^g C_{p,m}^o(T)$ had to, first, be determined. For this, we subtracted the function of $C_{p,m}^o(\mathrm{l},T)$ to $C_{p,m}^o(\mathrm{g},T)$. $C_{p,m}^o(\mathrm{l},T)$ consists of Equation (2), using the coefficients of Table 7. $C_{p,m}^o(\mathrm{g},T)$ was obtained by fitting the gas phase heat capacity data, computationally obtained through the B3LYP method in the temperature range from T = 280 K to T = 335 K, to a linear function:

$$C_{p,m}^o(\mathrm{g},T) = a + b\cdot(T/\mathrm{K}) \quad \text{(S6)}$$

The obtained a, b and c parameters are presented in Table S11.

Table S11. Coefficients of the linear fitting of the gas phase heat capacity values, obtained through the B3LYP method, as a function of temperature.

Ionic Liquid	T_{range} / K	a / J·K^{-1}·mol^{-1}	b / J·K^{-2}·mol^{-1}
[(SiC)C_1im][NTf_2]	280 to 335	159.61	1.0098
[(SiCSiC)C_1im][NTf_2]	280 to 335	193.16	1.2800
[(SiCSiCSiC)C_1im][NTf_2]	280 to 335	226.43	1.5464
[(Np)C_1im][NTf_2]	280 to 335	134.30	1.0081
[(Me_4C_5)C_1im][NTf_2]	280 to 335	142.32	1.2729
[(SiOSiC)C_1im][NTf_2]	280 to 335	199.58	1.2293

7. Computational quantum calculations

The following figures (S26 to S37) display all the stable conformers found for the different dimers. The energies are measured with respect to the minimum energy configuration of each dimer. Top and side-view are presented for each dimer.

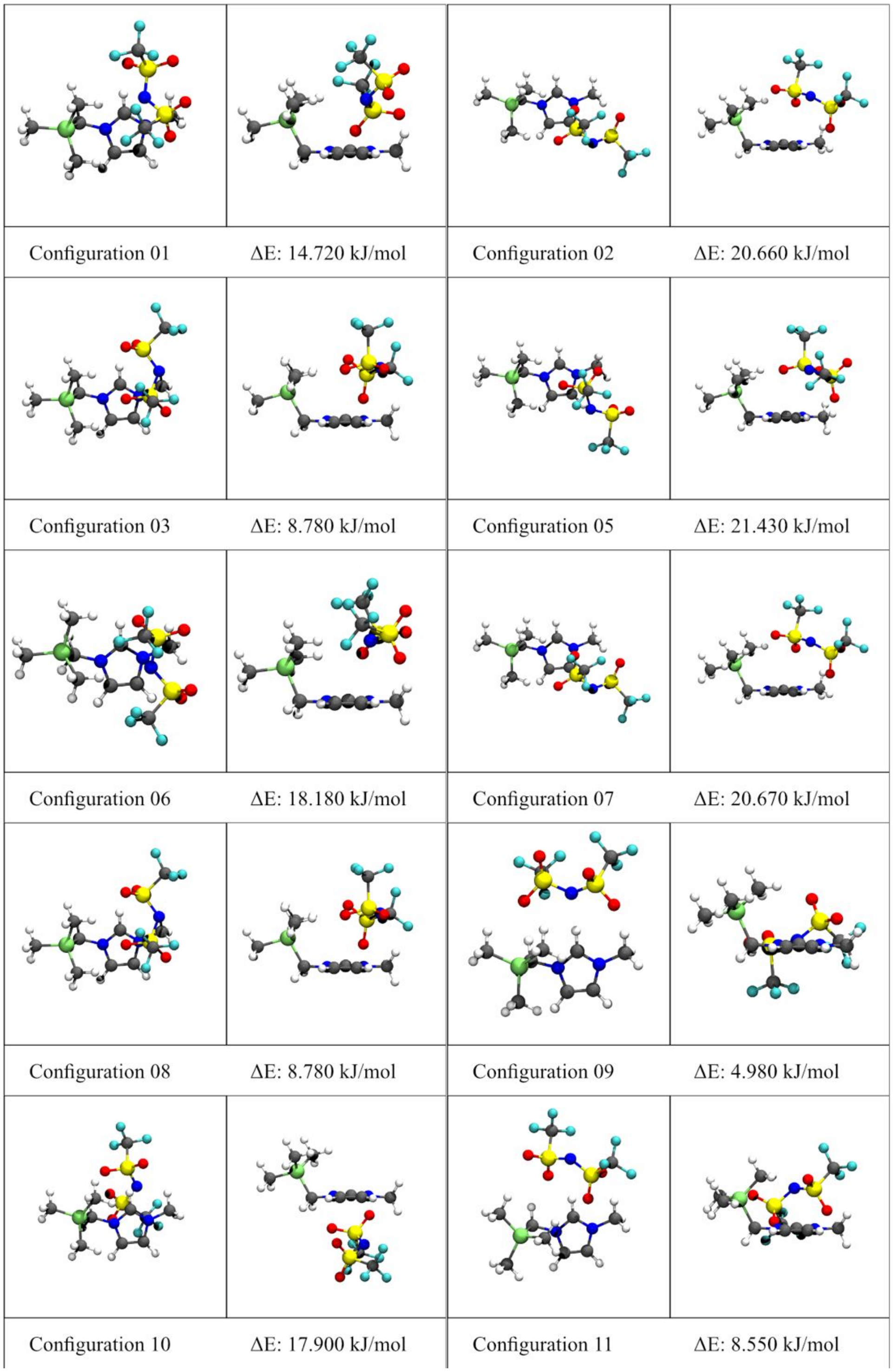

Figure S26. Stable configuration of the [(SiC)im][NTf_2] dimer (1/2).

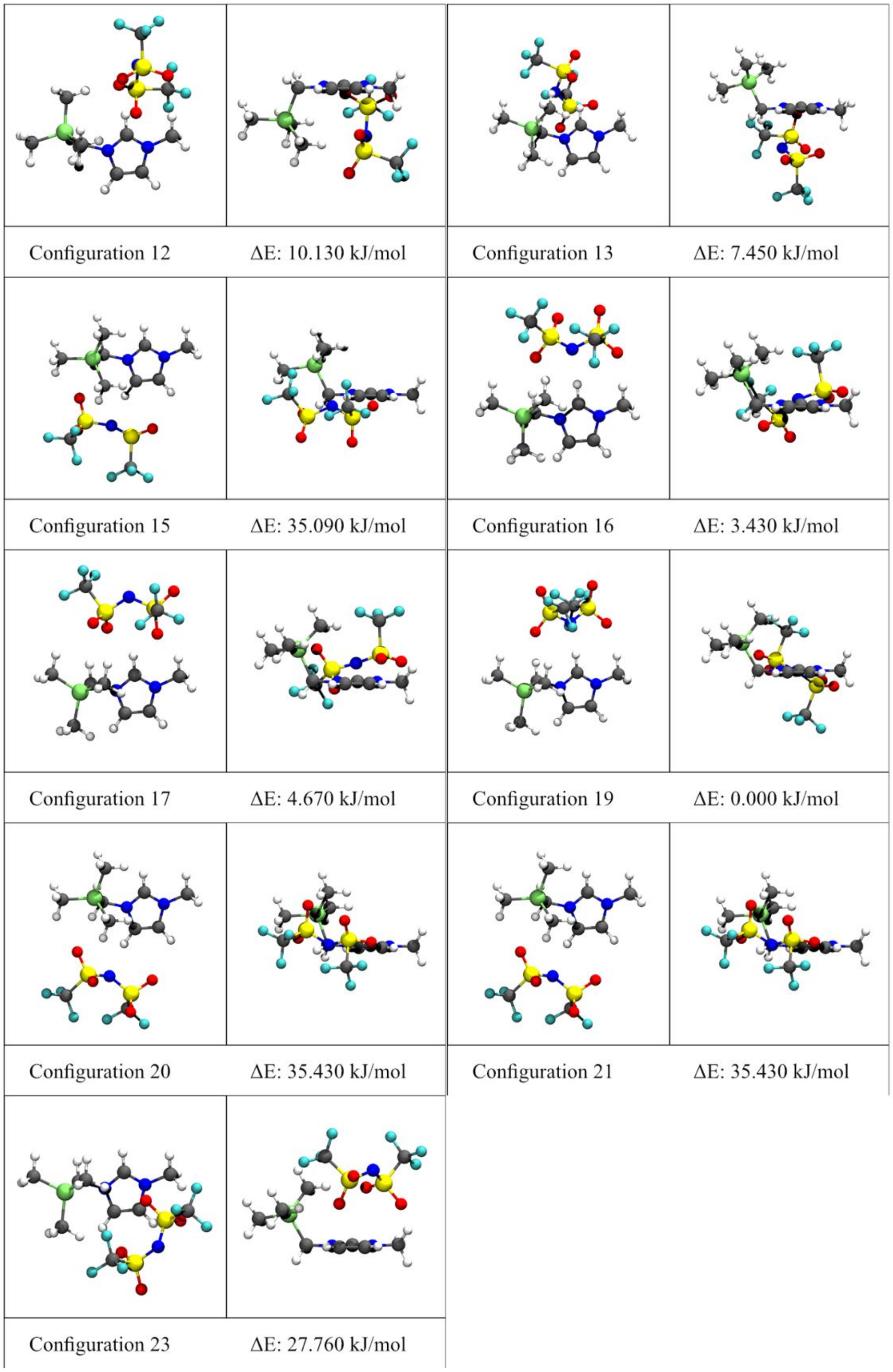

Figure S27. Stable configuration of the [(SiC)im][NTf_2] dimer (2/2).

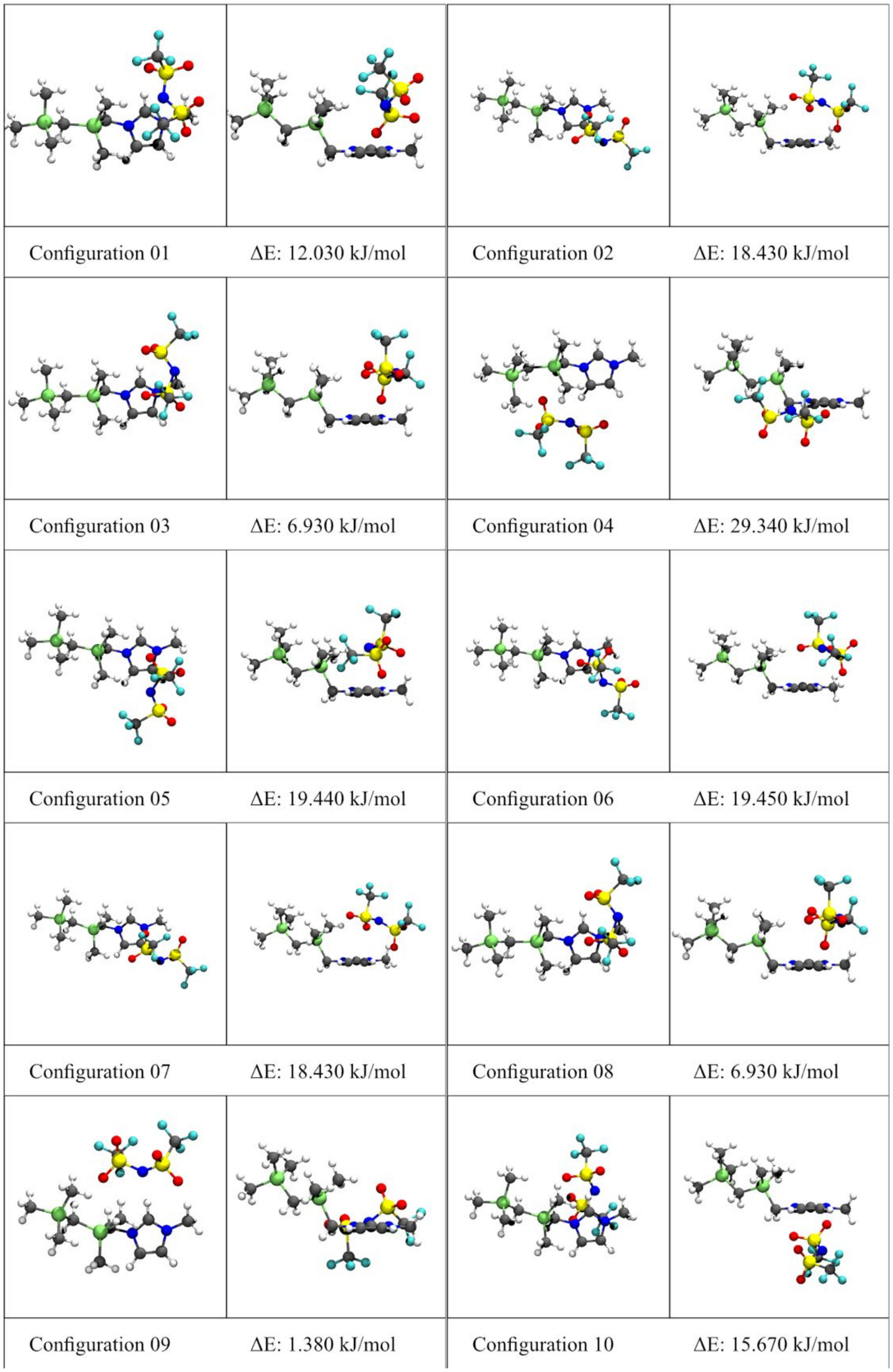

Figure S28. Stable configuration of the [(SiCSiC)im][NTf_2] dimer (1/2).

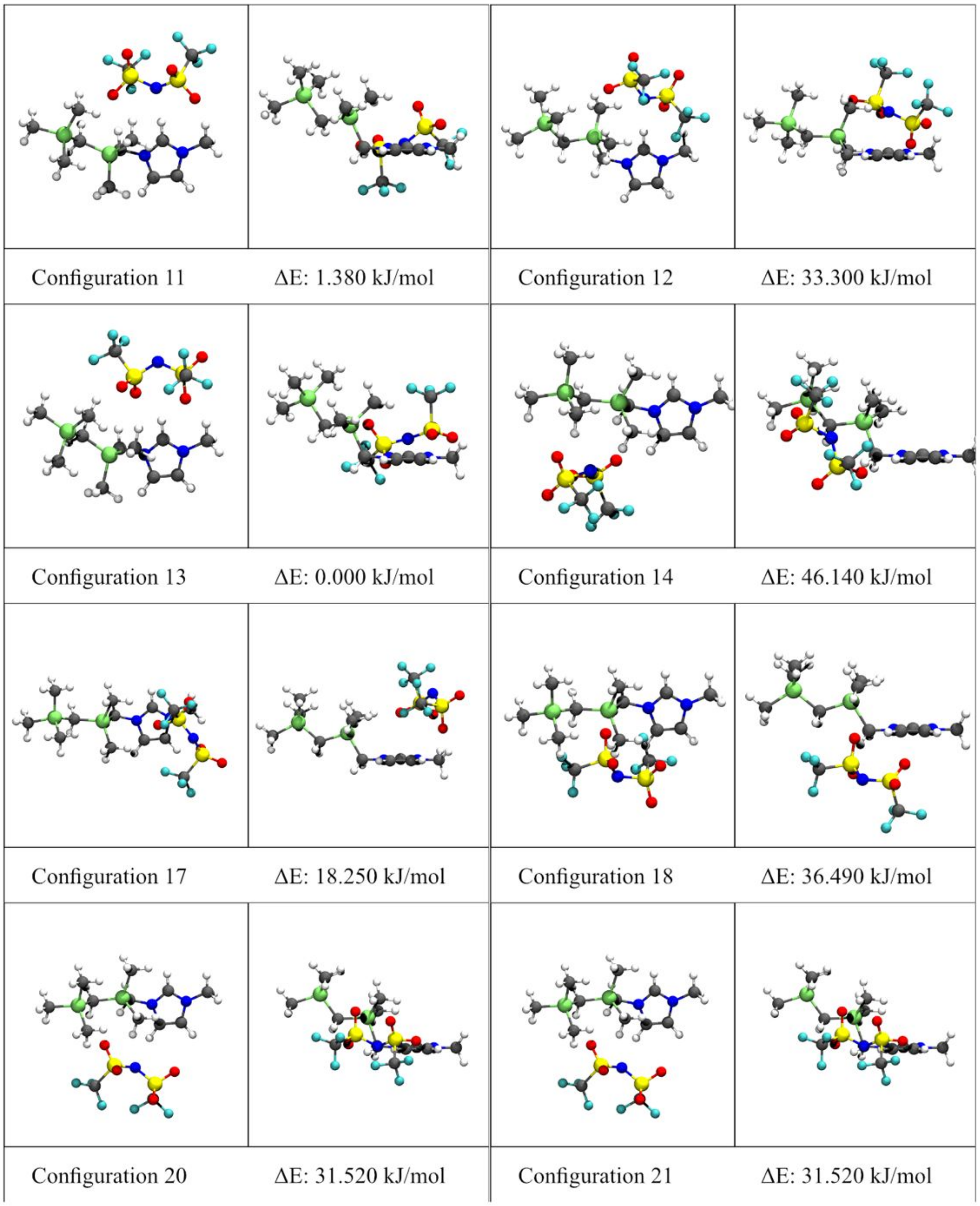

Figure S29. Stable configuration of the [(SiCSiC)im][NTf_2] dimer (2/2).

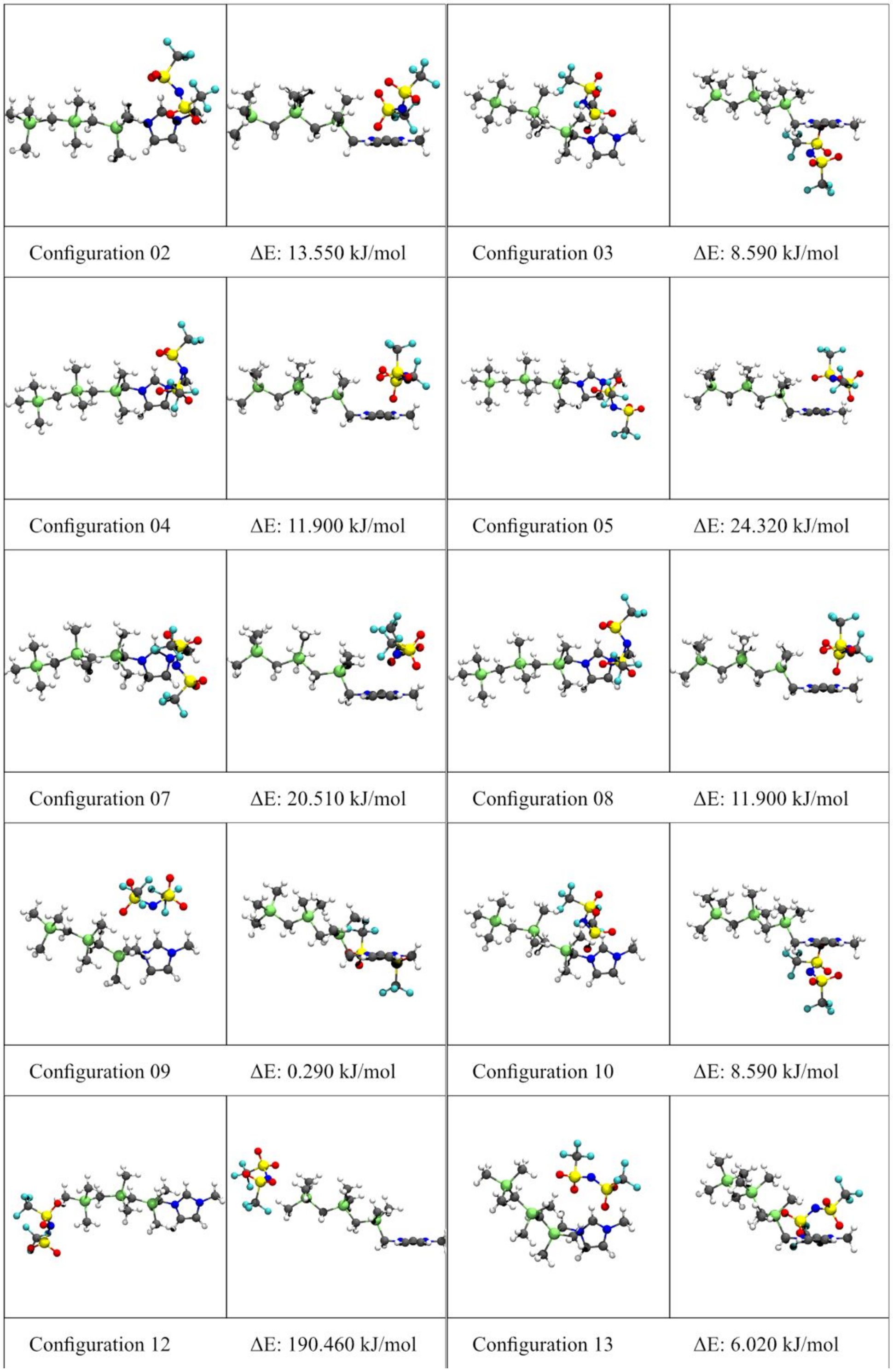

Figure S30. Stable configuration of the [(SiCSiCSiC)im][NTf_2] dimer (1/2).

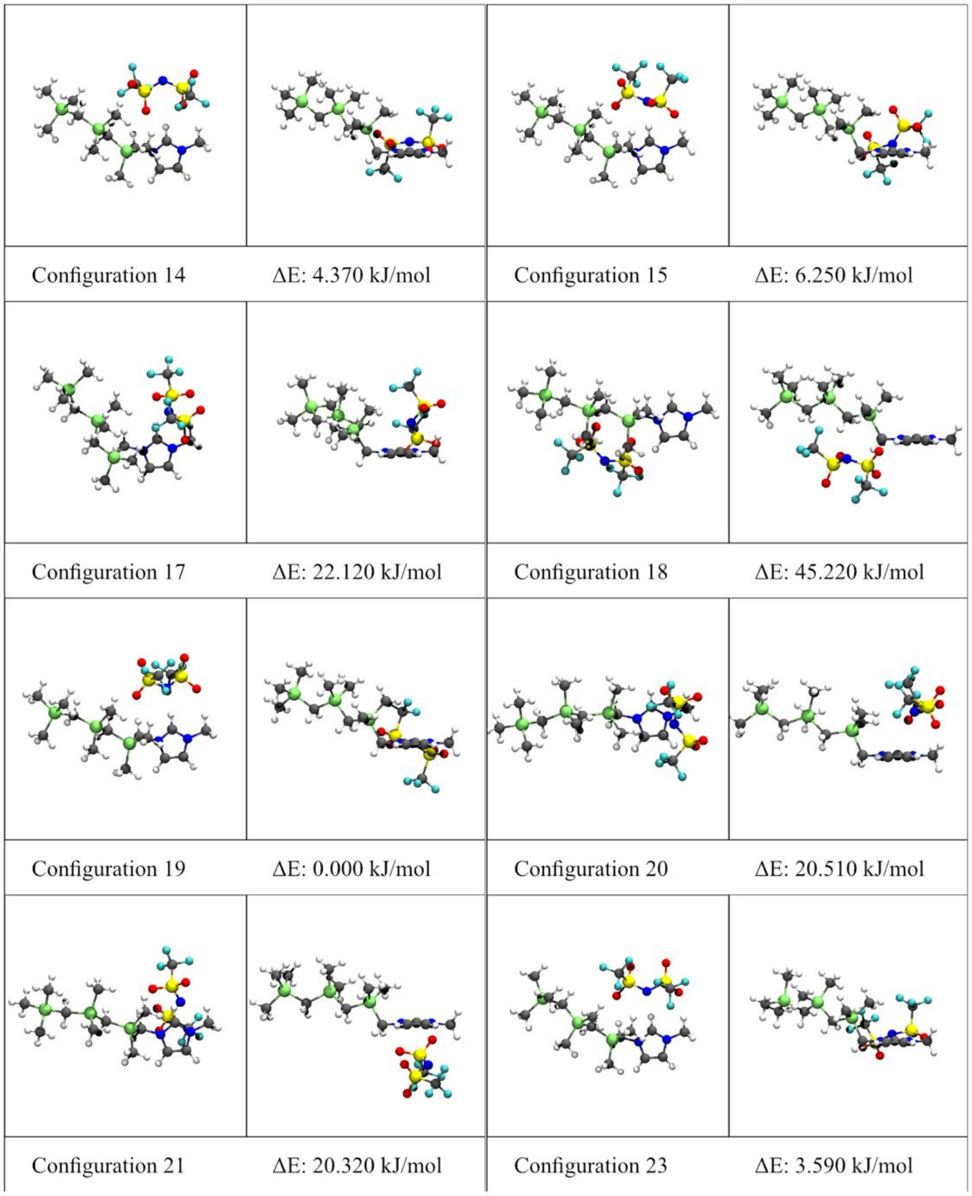

Figure S31. Stable configuration of the [(SiCSiCSiC)im][NTf_2] dimer (2/2)

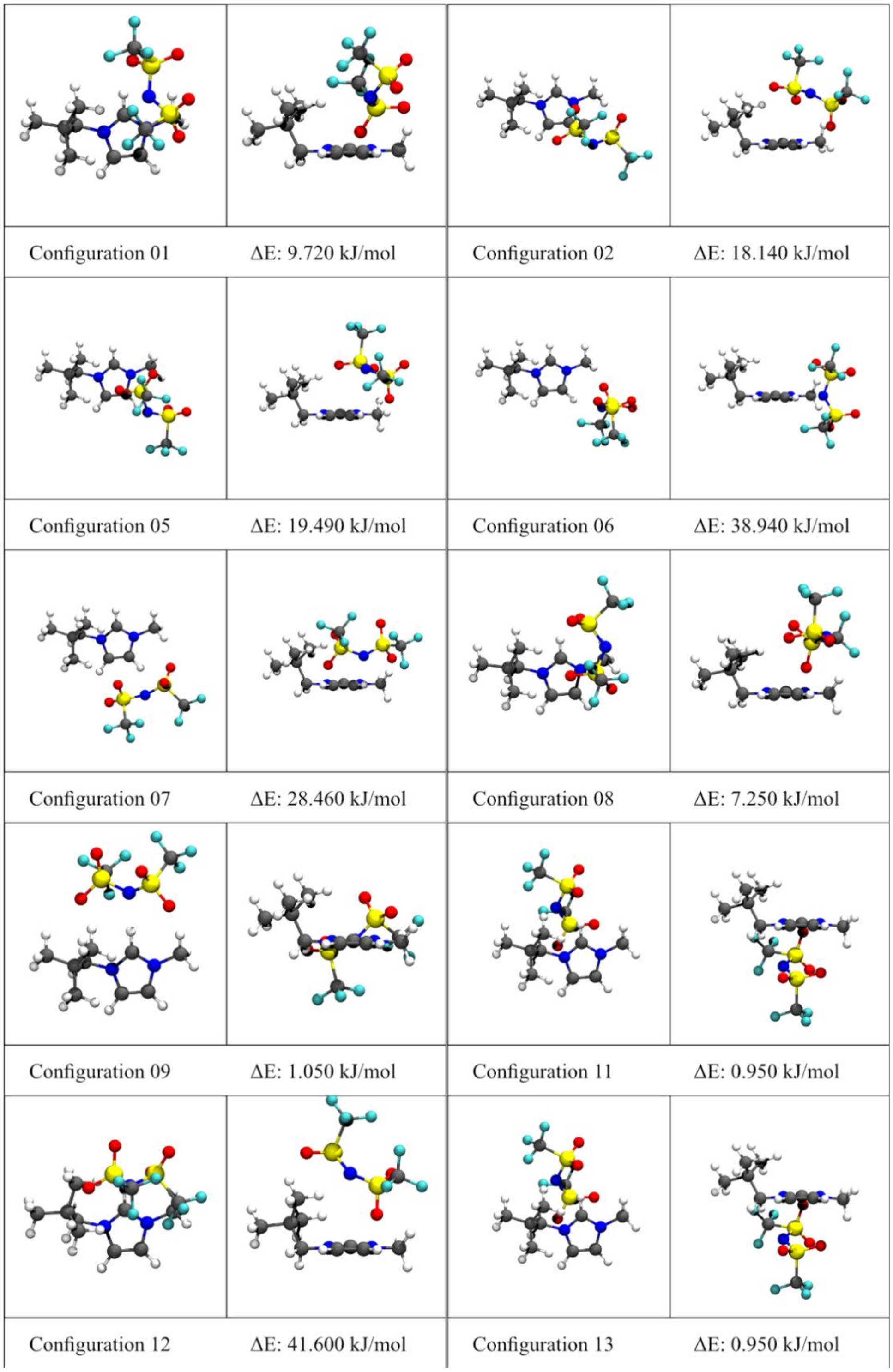

Figure S32. Stable configuration of the [(Np)im][NTf_2] dimer (1/2).

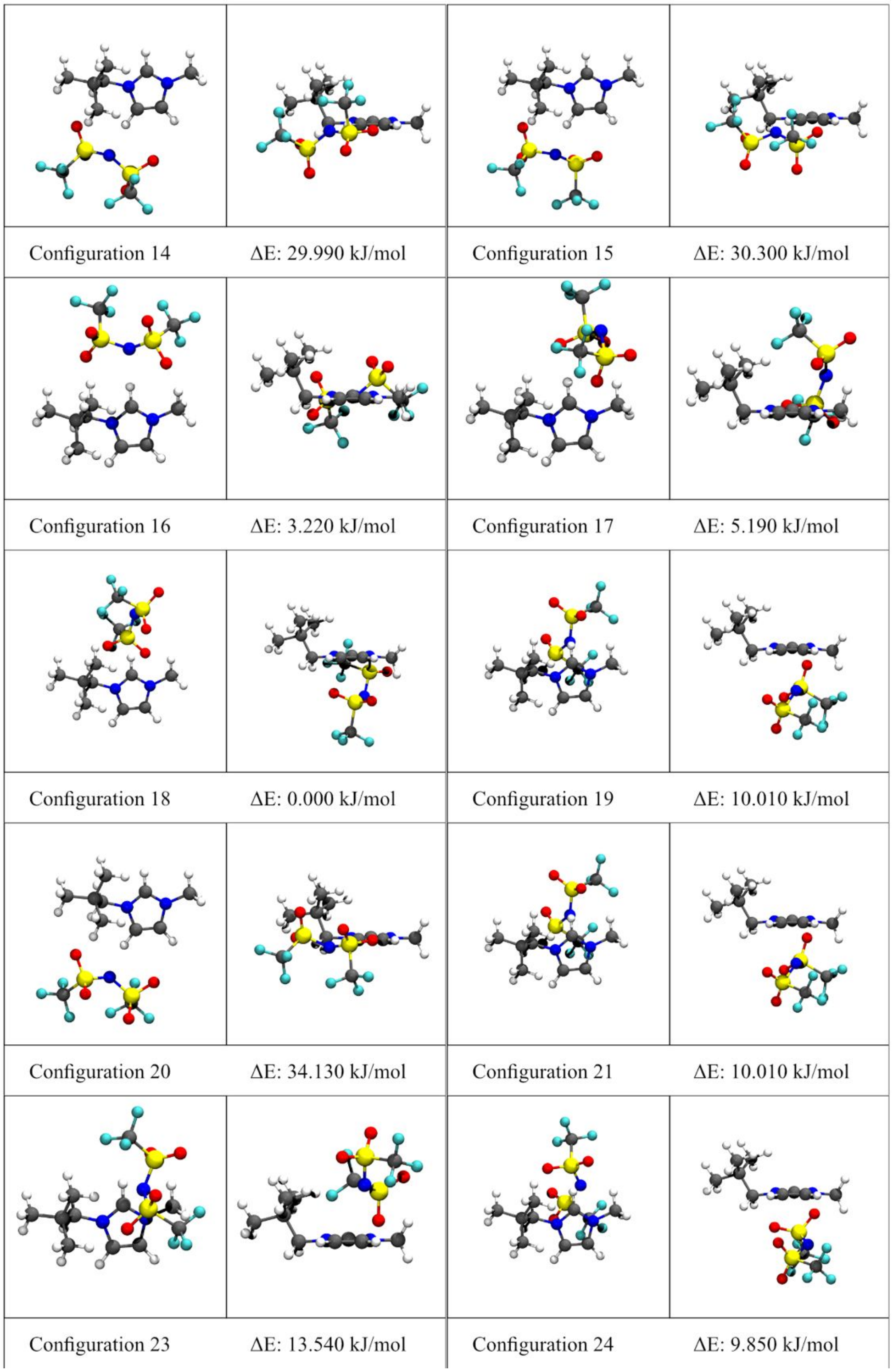

Figure S33. Stable configuration of the [(Np)im][NTf_2] dimer (2/2).

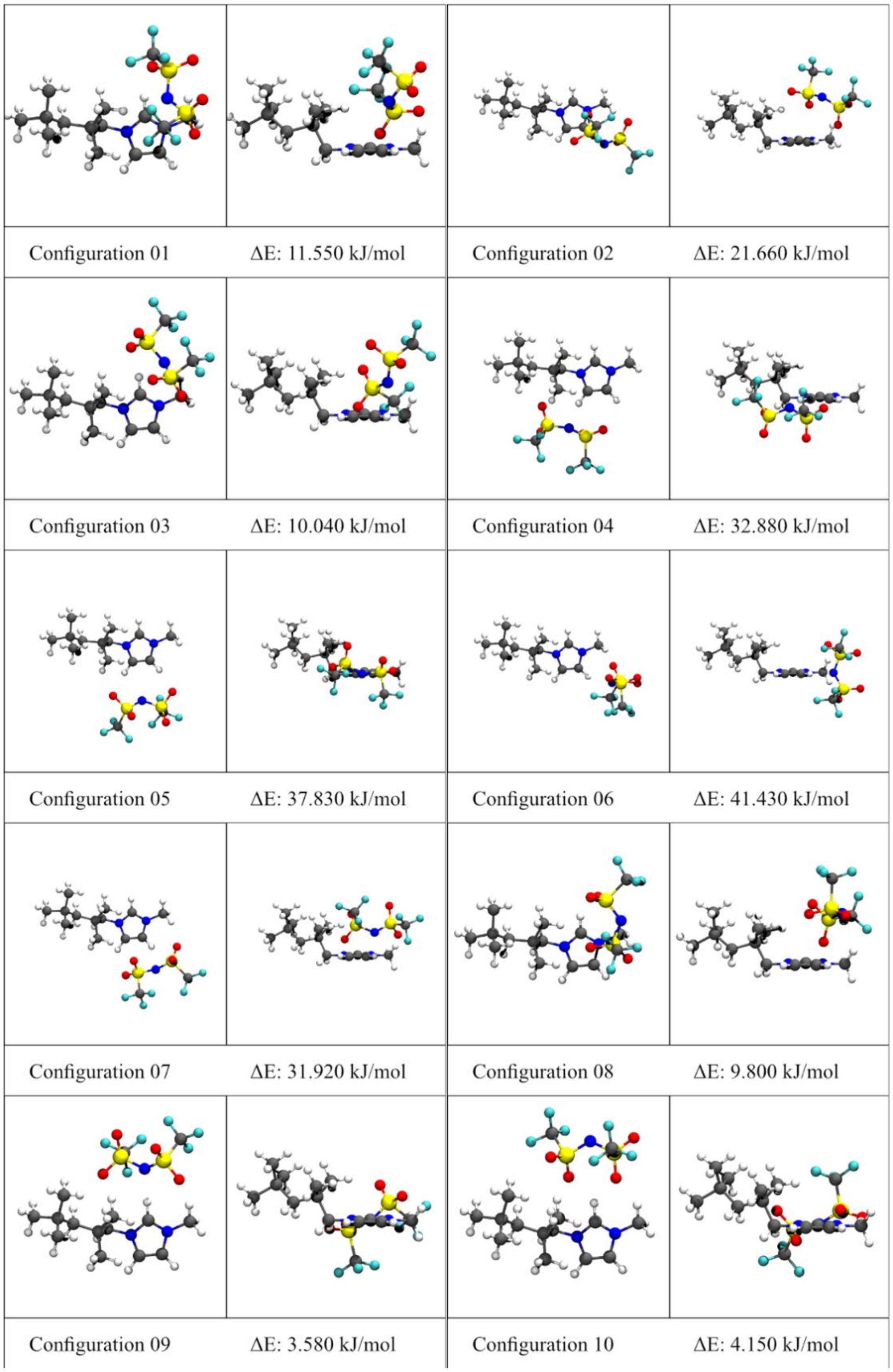

Figure S34. Stable configuration of the [(Me_4C_5)im][NTf_2] dimer (1/2).

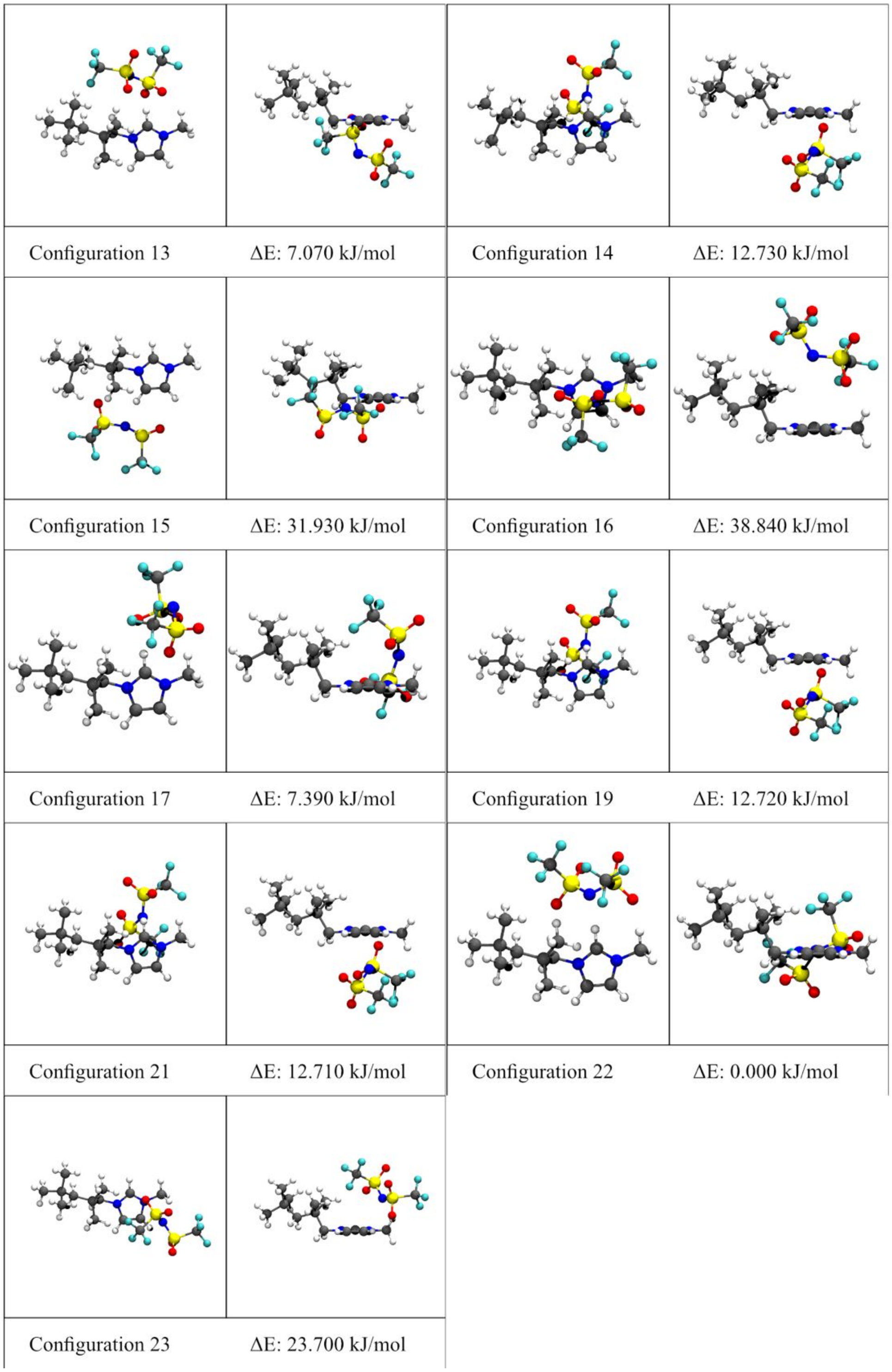

Figure S35. Stable configuration of the [(Me_4C_5)im][NTf_2] dimer (2/2).

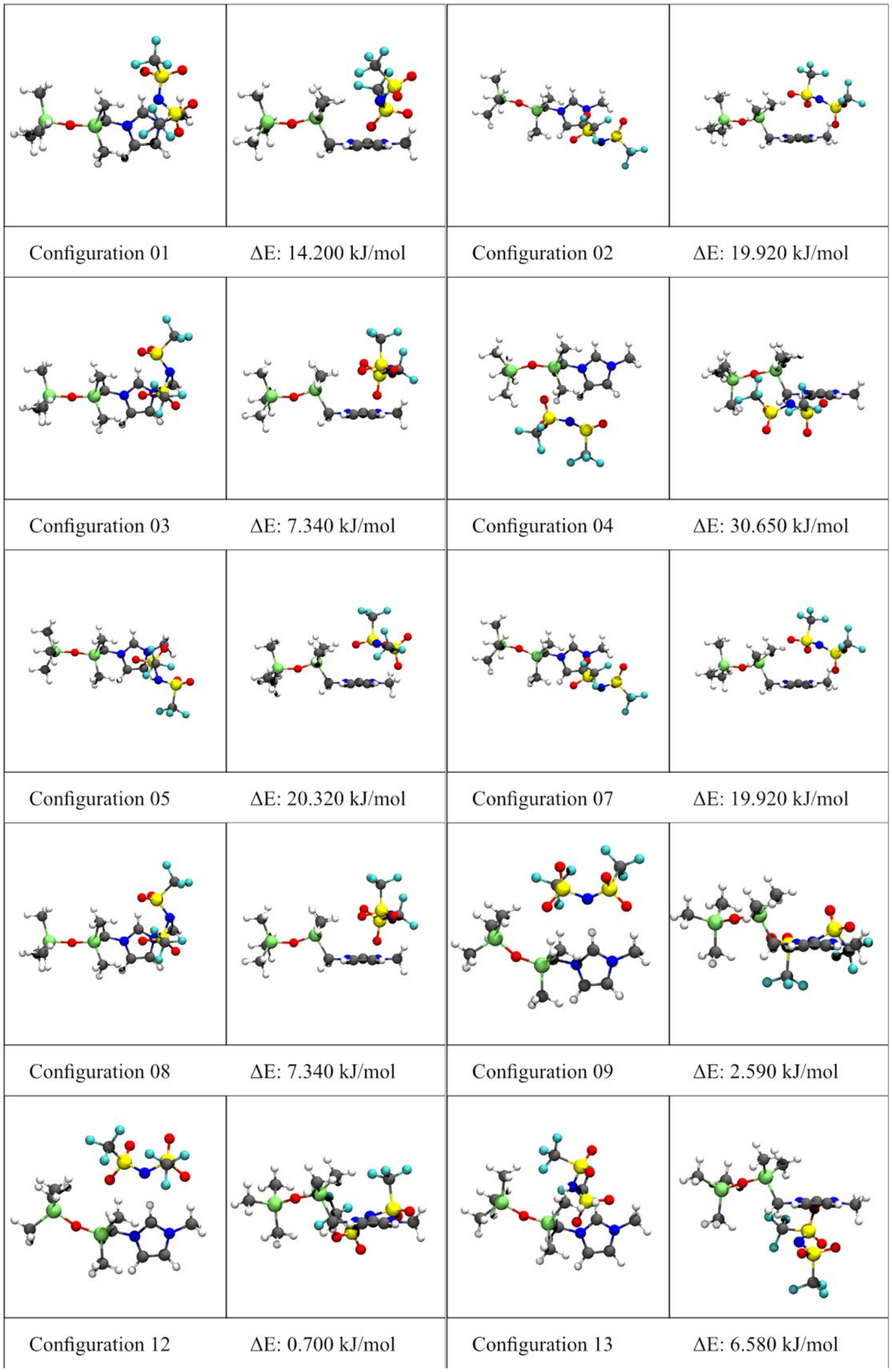

Figure S36. Stable configuration of the [(SiOSiC)im][NTf_2] dimer (1/2).

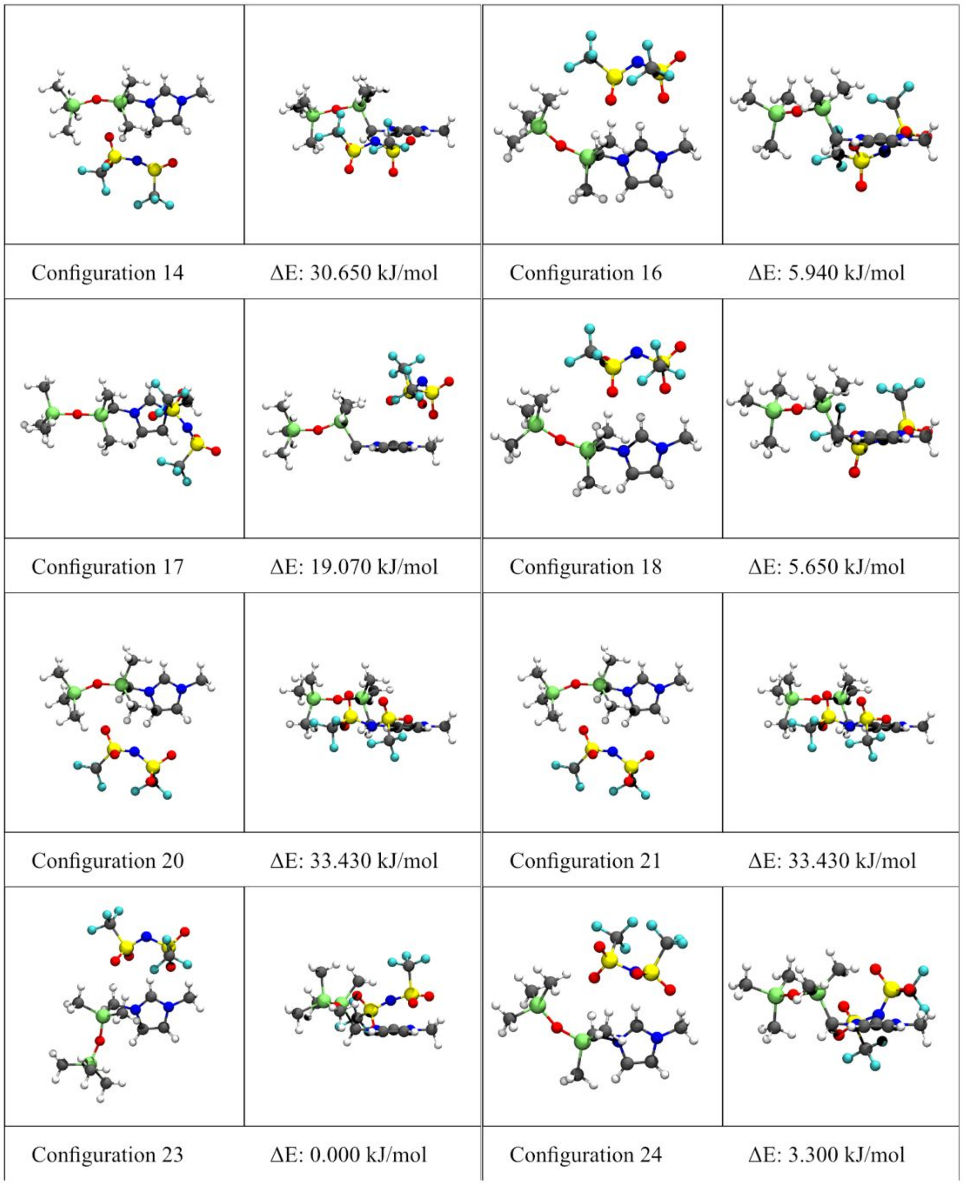

Figure S37. Stable configuration of the [(SiOSiC)im][NTf_2] dimer (2/2).

References

[1] R.D. Chirico, V. Diky, J.W. Magee, M. Frenkel, K.N. Marsh, Thermodynamic and thermophysical properties of the reference ionic liquid: 1-Hexyl-3-methylimidazolium bis[(trifluoromethyl)sulfonyl]amide (including mixtures). Part 2. Critical evaluation and recommended property values (IUPAC Technical Report), *Pure and Applied Chemistry*. **81** (2009) 791–828. https://doi.org/10.1351/PAC-REP-08-09-22.

[2] G.T. Furukawa, R.E. Mccoskey, G.J. King, Calorimetric properties of benzoic acid from 0-degrees to 410-degrees-K, *Journal of Research of the National Bureau of Standards*. **47** (1951) 256. https://doi.org/10.6028/jres.047.032.

[3] K. Moriya, T. Matsuo, H. Suga, Low-temperature adiabatic calorimeter with a built-in cryo-refrigerator, *The Journal of Chemical Thermodynamics*. **14** (1982) 1143–1148. https://doi.org/10.1016/0021-9614(82)90037-4.

[4] M. Sorai, K. Kaji, Y. Kaneko, An automated adiabatic calorimeter for the temperature range 13 K to 530 K The heat capacities of benzoic acid from 15 K to 305 K and of synthetic sapphire from 60 K to 505 K, *The Journal of Chemical Thermodynamics*. **24** (1992) 167–180. https://doi.org/10.1016/S0021-9614(05)80046-1.

[5] P. Goursot, H.L. Girdhar, E.F. Westrum, Thermodynamics of polynuclear aromatic molecules. III. Heat capacities and enthalpies of fusion of anthracene, *The Journal of Physical Chemistry*. **74** (1970) 2538–2541. https://doi.org/10.1021/j100706a022.

[6] M. Radomska, R. Radomski, Calorimetric studies of binary systems of 1,3,5-trinitrobenzene with naphthalene, anthracene and carbazole. I. Phase transitions and heat capacities of the pure components and charge-transfer complexes, *Thermochimica Acta*. **40** (1980) 405–414. https://doi.org/10.1016/0040-6031(80)80082-7.

[7] A. V. Blokhin, Y.U. Paulechka, G.J. Kabo, Thermodynamic properties of [C6mim][NTf2] in the condensed state, *Journal of Chemical and Engineering Data*. **51** (2006) 1377–1388. https://doi.org/10.1021/je060094d.

[8] X. Paredes, C.S.G.P. Queirós, F.J.V. Santos, A.F. Santos, M.S.C.S. Santos, M.J.V. Lourenço, C.A. Nieto De Castro, Thermophysical properties of 1-hexyl-3-methylimidazolium bis(trifluoromethylsulfonyl)imide, [C6mim][(CF3SO2)2N] - New data, reference data, and reference correlations, *Journal of Physical and Chemical Reference Data*. **49** (2020) 043101. https://doi.org/10.1063/5.0023160.

[9] M.A.A. Rocha, M. Bastos, J.A.P. Coutinho, L.M.N.B.F. Santos, Heat capacities at 298.15 K of the extended [C nC 1im][Ntf 2] ionic liquid series, *Journal of Chemical Thermodynamics*. **53** (2012) 140–143. https://doi.org/10.1016/j.jct.2012.04.025.
